# A Novel Nanoantibiotic Formulation With Magnetic and Targeting Potential: Ampicillin‐Conjugated l‐glu–Fe_3_O_4_ NPs

**DOI:** 10.1002/mbo3.70419

**Published:** 2026-09-27

**Authors:** Mehmet Demirel, Ozlem Baris, Mesut Taskin, Seyda Albayrak, Ferhunde Aysin, Azada Aliyeva, Buket Bakan

**Affiliations:** ^1^ Department of Molecular Biology and Genetics Faculty of Science, Atatürk University Erzurum Turkey; ^2^ Department of Biology Faculty of Science, Atatürk University Erzurum Turkey; ^3^ Department of Biology Science Faculty, Kyrgyz‐Turkish Manas University Bishkek Kyrgyzstan

**Keywords:** ampicillin, chemistry, drug delivery, magnetic nanoparticles, nanoantibiotics

## Abstract

Magnetic nanoparticles (NPs) are maintained in the body for a significantly longer duration than small molecule antibiotics, potentially facilitating prolonged therapeutic effects. In this study, a novel nanoantibiotic formulation (l‐glu–Fe_3_O_4_–Amp NPs) was prepared, and its antibacterial efficacy was evaluated. For this purpose, l‐glutamic acid (l‐glu) was used for surface modification of iron oxide nanoparticles (Fe_3_O_4_ NPs), and ampicillin (Amp) was then conjugated l‐glu–Fe_3_O_4_ NPs structure. Fourier transform infrared analyses revealed that the final nanoantibiotic formulation l‐glu–Fe_3_O_4_–Amp NPs was successfully prepared. The size distribution of the formulation was determined to be 164 nm by ZETAsizer. It exhibited a controlled drug release profile over a certain period of time, with a slightly increased release and then exhibited a stable profile. Agar well diffusion assay and the LIVE/DEAD BacLight fluorescent assay showed that the formulation had an antibacterial effectiveness against both *Bacillus cereus* and *Staphylococcus aureus*. The formulation has a lower minimal inhibitory concentration value against *S. aureus*. It significantly increased the accumulation of reactive oxygen species in both bacteria when compared with the Amp group. Its cellular uptake and bacterial interaction were confirmed by confocal and transmission electron microscopy images. The in vitro assay revealed that this formulation is nontoxic to fibroblast cells. This study highlights the potential of l‐glu–Fe_3_O_4_–Amp NPs conjugate as a potentially effective antibacterial agent. This is the first report on the use of l‐glu as a surface modifier of Fe_3_O_4_ NPs and Amp. Use of l‐glu increases the drug‐carrying capacity of NPs and reduces the possible side effects of Amp.

## Graphical abstract

A novel nanoformulation consisting of ampicillin (Amp), l‐glutamic acid (l‐glu), and iron oxide nanoparticles was prepared. l‐glu–Fe_3_O_4_–Amp NPs exhibited a controlled drug release profile over a certain period of time. The antibacterial effectiveness of the formulation was proven by agar well diffusion and the LIVE/DEAD BacLight fluorescent assays. The formulation led to the formation of reactive oxygen species in the test bacteria. The nanoformulation is nontoxic to fibroblast cells.

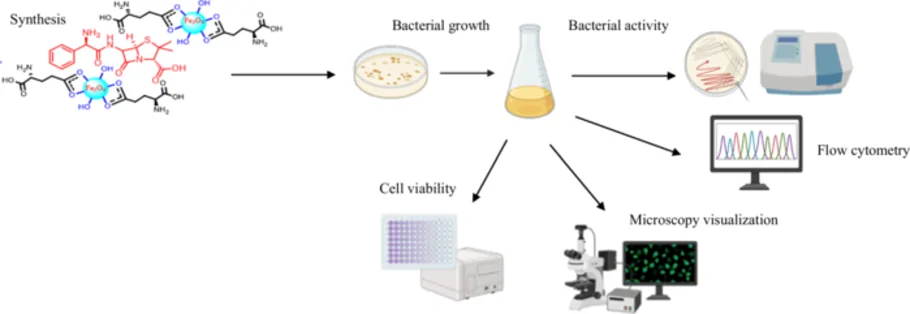

## 1 Introduction

Nanomedicines have demonstrated efficacy in the treatment of severe health disorders, such as cancer, hepatitis, and diabetes. These offer numerous advantages, including enhanced tissue absorption, selective mechanisms of action, and improved intracellular targeting (Farooq et al. 2019). Magnetic nanoparticles (NPs) are utilized in significant biological applications, such as drug delivery, biomagnetic separation, and detection of cells, proteins, nucleic acids, enzymes, and microorganisms (Binandeh and Karimi 2018).

Nanoantibiotics are one of the strategies being investigated to prevent the proliferation of antibiotic‐resistant bacteria. As carriers and delivery agents, these materials can reach target sites within a bacterium by crossing the membrane, interacting with cellular components, and exhibiting metabolic activity (Landa et al. 2025). Advances in nanoantibiotic delivery technologies facilitate the effective administration of antibiotics by enhancing pharmacokinetics and accumulation while attenuating drug‐induced adverse effects on healthy cells. The administration of antibiotics via an NP formulation presents numerous benefits, such as manageable and consistent distribution throughout the target tissue, reduced frequency and dosage, enhanced solubility, prolonged and regulated release, and reduced adverse effects (Huh and Kwon 2011).

Iron oxide nanoparticles (Fe_3_O_4_ NPs) have garnered significant attention for their biomedical applications, including magnetic resonance imaging, cancer therapy, antibiotic delivery, and biomolecule separation (Rashid et al. 2021). Additionally, surface modifications with these particles are made to enable the attachment of more functional groups to the structure in drug delivery research. Therefore, many initiatives have been undertaken to coat and modify the surfaces of Fe_3_O_4_ NPs using diverse materials, including polyethylene glycol, silica, amino acids, and ligand molecules (Wu et al. 2008). For instance, l‐glutamic acid (l‐glu), a biocompatible nonessential amino acid, could be used to modify the surface of the Fe_3_O_4_ NPs‐doxorubicin conjugate (Dutta et al. 2020). On the contrary, there is no study on the use of l‐glu for surface modification of Fe_3_O_4_ NPs‐antibiotic conjugates.

In the literature, it has been documented that l‐glu can increase the binding potential of drugs, since its amine groups can interact with the hydroxide functional groups on the Fe_3_O_4_ surface (Dutta et al. 2020). In this way, a conjugate of l‐glu and Fe_3_O_4_ can enhance antibiotic delivery, improve their therapeutic efficacy at even lower doses, and reduce their potential toxic effects. For example, it can be concluded that using a conjugate of l‐glu–Fe_3_O_4_–ampicillin (Amp) enables controlled Amp release, increases its therapeutic efficacy, and reduces its potential toxic effects.

This study aims to (1) produce a novel nanoantibiotic formulation consisting of l‐glu, Fe_3_O_4_ NPs, and Amp, (2) investigate its antimicrobial effectiveness against bacteria, and (3) evaluate its cytotoxicity on fibroblasts. This is the first attempt at the use of l‐glu as a surface modifier in the preparation of a conjugate of Fe_3_O_4_ NPs and Amp.

## 2 Materials and Methods

### 2.1 Materials

Fe_3_O_4_ NPs were obtained commercially from *Nanografi* Company with primary sizes of 18–28 nm and 98.45% purity. Premixed Water‐Soluble Tetrazolium 1 (WST‐1) reagent was purchased from Takara Co. All other chemicals were supplied by Sigma Chemical Co. (St. Louis, MO, USA).

### 2.2 Synthesis of l‐glu‐Coated Fe_3_O_4_–Amp NPs

Surface modification with l‐glu and synthesis of nanoformulation were carried out according to Mallakpour and Madani (2015) and Binandeh and Karimi (2018) with minor modifications. First, l‐glu (15 wt%) was dissolved in methanol (20 mL), then 0.10 g of Fe_3_O_4_ NPs was added, and the mixture was left on the magnetic stirrer for 24 h at room temperature. The final mixture was placed in an ultrasonic bath for 30 min. The suspension containing l‐glu–Fe_3_O_4_ was filtered (0.22 µm membrane filter) and then washed three times with methanol to remove unbound particles. An amount of 350 mg Amp in water was mixed with 20 mg of l‐glu–Fe_3_O_4_, and the prepared solution was then left to react at room temperature (25°C) under magnetic stirring for 24 h. After incubation, the solution was washed three times with deionized water to remove unreacted Amp. The prepared final material (l‐glu–Fe_3_O_4_–Amp) was kept at +4°C until use.

### 2.3 Characterization Analyses

The characterization of l‐glu–Fe_3_O_4_–Amp was conducted according to Fourier Transform Infrared (FTIR) Spectroscopy (Bruker VERTEX 70v), ZETAsizer (Malvern ZETAsizer Nano ZSP), Scanning Electron Microscopy (SEM, SHIMADZU UV‐1800), and energy‐dispersive X‐ray (EDX) spectroscopy. In FTIR spectra, the samples were dried at room temperature, and spectra were collected over the range of 4000–500/cm in transmittance mode. For ZETA measurements, the samples were dispersed in relevant solutions (at different pH and fetal bovine serum [FBS]) at a concentration of 0.1 mg/mL by sonication for 10 min to obtain a homogeneous suspension. In the SEM analyses, the dried samples were mounted onto aluminum stubs using double‐sided conductive carbon tape, and then the samples were sputter‐coated with a thin layer of gold to improve electrical conductivity prior to imaging.

### 2.4 Drug‐Loaded Capacity and In Vitro Release Profile

The drug‐loading (DL) capacity of l‐glu–Fe_3_O_4_ NPs was evaluated according to the previously described method (Ardekani et al. 2019). Amp and l‐glu–Fe_3_O_4_ NPs were mixed at equal volumes (1 mg/mL) and the suspension was then stirred at the dark and 25°C for 2 h. The suspension was centrifuged at 5000*g* for 20 min, and the resulting supernatant was used to determine the unbound antibiotic. The amount of Amp in the supernatant was determined at 268 nm by ultraviolet–visible (UV–Vis) Spectrophotometer. The encapsulation efficiency and DL efficiency (DL %) were calculated according to the formula below (Li et al. 2018):

EE (%)=original drug added (mg)−drug remained in supernatant (mg)/original drug added (mg)∗100,

DL (%)=weight of drug in formulation/weight of nanoparticle containing∗100.

An in vitro release profile was performed according to a standard protocol. In brief, 1 mL of distilled water is added to the l‐glu–Fe_3_O_4_–Amp and placed in a dialysis bag inside a beaker containing ~50 mL of phosphate‐buffered saline (PBS) (pH 7.4). At certain time intervals (1, 2, 3, 4, 6, 12, and 24 h), 1 mL of dissolution medium is taken from the beaker, and the same amount of fresh PBS is added. Subsequently, the concentration of the released drugs is measured by UV–Vis spectrometry at certain time intervals. The cumulative release was plotted against time, and the release profiles were adapted to three kinetic models (zero‐order, Higuchi, and Korsmeyer–Peppas) to understand the release mechanism. The following equations were used (Mariz et al. 2022; Elmotazbellah et al. 2025).

*Zero‐order model:*

$$Q_{t}=Q_{0}+K_{0}t,$$

where *Q_t_* is the amount of drug in time *t*, *Q*_0_ is the initial amount of drug in the solution, and *K*_0_ is the zero‐order release constant.

*Higuchi model:*

$$f_{t}=K_{H}t^{1/2},$$

where *f_t_* is the fractional release of the drug and *K*_H_ is the Higuchi dissolution constant.

*Korsmeyer–Peppas model:*

$$M_{t}/M_{\infty }{=K}_{\text{sp}}t^{n},$$

where *M_t_*/*M*_∞_ is the fractional release of the drug, *n* is the release exponent that indicates the drug release mechanism, and *K*_sp_ is the constant that incorporates the structural modifications.

### 2.5 Molecular Docking Analyses

Penicillin‐binding protein (PBP) was selected as the docking target because it is a cell wall–associated enzyme and the primary molecular target of β‐lactam antibiotics such as Amp in Gram‐positive bacteria, allowing assessment of whether the l‐glu–Fe_3_O_4_–Amp formulation retains its ability to interact with bacterial cell wall–related targets (Miyachiro et al. 2019).

The two‐dimensional structure of l‐glu–Fe_3_O_4_–Amp ligand was drawn using ChemDraw 20.0 and saved in Structure Data File format (S. Kim et al. 2023). The structure of the Amp ligand (PubChem CID: 6249) was retrieved from PubChem database (https://pubchem.ncbi.nlm.nih.gov). The crystal structure of PBP1a (PDB ID: 5TRO) was retrieved from the Protein Data Bank (https://www.rcsb.org) (Berman 2000). Then, the molecular docking analysis of PBP1a protein with l‐glu–Fe_3_O_4_–Amp ligand was performed by CB‐DOCK server (http://clab.labshare.cn/cb-dock/php/blinddock.php) (Liu et al. 2022). Finally, the protein–ligand docked structure was shown and colored by the Chimera 1.14 visualization tool (Pettersen et al. 2004).

### 2.6 Evaluation of Antibacterial Effectiveness

#### 2.6.1 Agar Well Diffusion Assay

The antimicrobial activities of the test samples were assessed against two Gram‐positive bacteria, namely *Bacillus cereus* (ATCC 11778) and *Staphylococcus aureus* (ATCC 25923), using the agar well diffusion assay. Test organisms were grown in tryptic soy broth (TSB) for 16–24 h at 37°C. They are diluted with isotonic saline solution (NaCl 0.9%) to obtain a turbidity of 0.5 McFarland standard (⁓10^8^ CFU/mL). The prepared bacterial suspensions were spread uniformly on tryptic soy agar. Wells of 6 mm were opened on each agar plate, where spread culture was made using a cork borer (cork borer no: 3) and 40 μL of controls (Positive control‐Negative control) and test samples were added to the relevant wells. After incubating the culture plates at 37°C for 24 h, the inhibition zones surrounding the wells were measured and assessed as having a diameter of mm (Sreedharan and Singh 2019).

#### 2.6.2 Determination of Minimal Inhibitory Concentration (MIC)

The study was based on the National Committee for Clinical Laboratory Standards: Methods for Dilution Antimicrobial Susceptibility Tests for Bacteria that Grow Aerobically; Approved Standard—Eighth edition. NCCLS document M7‐A6. It was carried out within the scope of the “NCCLS” standard. Microorganisms that showed activity against test samples by the agar well diffusion method were used in the test. Test organisms were grown in TSB for 16–24 h at 37°C. They were diluted with isotonic saline solution to obtain a turbidity of 0.5 McFarland standard. The concentration of test samples was adjusted to 500–7.8 µg/mL. After 20 s of mixing at 300 rpm, the plate was incubated at 37°C for 24 h (Güllüce et al. 2004). All other microbial analyses were conducted at concentrations selected based on the MIC values.

#### 2.6.3 Growth Kinetic Analysis

Culture growth curves of *B. cereus* and *S. aureus* were followed and examined in the absence or presence of test samples. Main cultures of the test organisms were inoculated into TSB with 1/10 of the specified culture gradient and incubated at 37°C and 120 rpm for 24 h. Cultures were prepared by determining the test sample at concentrations with no application, and the effect was observed as a control. The sterile stock solution of the test samples was created by dispersing them in sterilized TSB and subjecting them to sonication for a duration of 10 min. A volume of 30 μL of bacterial parent culture was incorporated into the various reaction mixtures set up in a 96‐well plate, and the reaction volumes were modified by adding nutrient broth to achieve a final volume of 300 μL. Growth kinetic studies were conducted by assessing the optical density (OD) at 600 nm with a plate reader at consistent time intervals. At approximately the midpoint of the logarithmic phase of bacterial growth (between the 8th and 10th hours), the test sample concentrations were added to their respective reaction mixtures. Following the addition of the samples, data collection for the growth curve commenced after a 10‐min period of inactivity (Arakha et al. 2015).

#### 2.6.4 LIVE/DEAD BacLight Fluorescence Test

This application serves as a significant method for visualizing the antimicrobial effects of test samples, particularly NPs, in a culture. To differentiate live from dead cells, bacterial cells (control) and those treated with the concentrations obtained from the MIC test were used with the LIVE/DEAD BacLight bacterial viability kit on a flow cytometer (Beckman Coulter CytoFLEX).

Cultures of *B. cereus* and *S. aureus* were established in distinct bottles by introducing 1 mL of overnight culture into TSB medium. In the mid‐log phase of bacterial growth, the test samples were added, and the cultures were allowed to grow until the late log phase. From these cultures, 25 mL of each bacterial solution was taken and centrifuged at 7000 rpm for 15 min. The liquid fraction (supernatant) was removed, and the obtained pellets were suspended in 2 mL of HEPES buffer (10 mM, pH 7.4, with 150 mM NaCl). One mL of the prepared bacterial suspensions was introduced into 20 mL of HEPES buffer across two distinct tubes. The samples were incubated at room temperature for 1 h, with mixing every 15 min. The suspensions underwent centrifugation at 7000 rpm for 15 min. Ultimately, the pellets were resuspended in 10 mL of HEPES buffer in separate tubes, and the OD value was assessed at 670 nm. A total of 3 μL of the dye mixture, consisting of equal volumes of SYTO9 and propidium iodide (PI) dyes, was introduced into each 1 mL of the prepared bacterial samples. Following thorough mixing, the bacterial suspensions were incubated in the dark for a duration of 15 min. Flow cytometry analysis was used to capture images of green fluorescence, indicating live cells, and red fluorescence, indicating dead cells. The excitation and emission wavelengths are set at 480/500 nm for SYTO9 and 490/635 nm for PI. The ratio of red to green volume reflects the proportion of dead cells in each sample (Arakha et al. 2015; Balto et al. 2020).

#### 2.6.5 Analysis of Oxidative Stress (Reactive Oxygen Species [ROS]) Level

The DCFH‐DA test is an oxidative stress test using a fluorescent molecular probe. It is based on the oxidation of 2′,7‐dichlorofluorescein (DCFH) dye by different ROSs (Keteci and Bakan 2025). DCFH‐DA readily traverses cell membranes and is hydrolyzed by cellular esterases to produce DCFH, which is then oxidized to the fluorescent compound 2′,7′‐dichlorofluorescein (DCF) in the presence of hydroxyl radicals (Beus et al. 2023).

In this study, ROS formation was measured in bacterial cells, and the concentrations were set according to the MIC value. Following a 24‐h exposure, the cells underwent three washes with 1× PBS and were subsequently stained with 20 μM DCFH‐DA dye for a duration of 30 min. The cells were then washed twice more with PBS and analyzed using a plate reader at a wavelength of 485 nm and an emission wavelength of 535 nm. Data are expressed as percent fluorescence compared with corresponding negative controls (Arakha et al. 2015).

### 2.7 Investigation of In Vitro Cytotoxicity

L929 (fibroblast cell line, ATCC CCL‐1) cells were cultured in DMEM/F‐12 medium containing l‐glutamine, 10% FBS, 100 U/mL penicillin/streptomycin supplements at 37°C in a 5% CO_2_ incubation. The cytotoxicity was determined by WST‐1 test which a colorimetric assay dedicated to cell viability (Bakan et al. 2020). For this purpose, the cells (2 × 10^4^ cells) were initially cultured in a 96‐well plate, and after 24 h, they were treated with the formulation (l‐glu–Fe_3_O_4_–Amp) or Amp alone at the different concentrations (6.25–500 μg/mL) and then were incubated for 24 h at 37°C.

The concentrations were selected based on preliminary studies for cell culture systems, which are commonly used in toxicological research to explore dose‐dependent cellular responses. Untreated cells were employed as a negative control. The OD was measured at 450 nm in a plate reader. Relative viability was assessed against the negative controls (untreated cells). The cell viability was calculated as follows:

(%) viable cells=([(absorbance of treated cells)–(absorbance of the blank)])/([(absorbance of the control)–(absorbance of the blank))×100.

### 2.8 Transmission Electron Microscopy (TEM) Imaging

Bacterial cultures were prepared in separate bottles by inoculating the overnight culture. In the mid‐log phase of bacterial growth, test samples were added at the concentrations obtained from the MIC test and allowed to grow until the late log phase. An amount of 25 mL of each bacterial solution from these cultures was centrifuged at 7000 rpm for 15 min. The supernatant was discarded, and the pellets were resuspended in PBS. The OD was measured at 670 nm prior to TEM imaging.

### 2.9 Cellular Internalization in Bacteria

l‐glu–Fe_3_O_4_–Amp was fluorescently labeled with minor modification of the study of (Solak et al. 2025). First, 1 mg of Oregon Green 488‐X was dissolved in 0.2 mL of dimethylformamide, and the solution was stirred in the dark for 1 h. The amount of 0.4 mL of dye solution was mixed with l‐glu–Fe_3_O_4_–Amp (1.6 mL). After the incubation period, the mixture was centrifuged and kept in the dark until use. For the cell internalization study, bacterial cells were treated with Oregon Green 488‐X‐labeled l‐glu–Fe_3_O_4_–Amp and after the washing step, cells were observed and recorded under confocal microscopy by measuring at 610 and 623 nm.

### 2.10 Statistical Analysis

Statistical analyses were conducted utilizing GraphPad Prism 8 (GraphPad Software, LLC, Boston, MA, USA), and the results were compared with the control group through one‐way analysis of variance. All values are presented as means ± standard deviation (SD). A statistical significance threshold was established at *p* < 0.05.

## 3 Results

### 3.1 Synthesis and Characterization of l‐glu‐Coated Fe_3_O_4_–Amp

To prepare the nanoantibiotic formulation, l‐glu was used for surface modification of Fe_3_O_4_ NPs, and Amp was then conjugated l‐glu–Fe_3_O_4_ NPs structure. The final formulation (l‐glu–Fe_3_O_4_–Amp NPs) were schematically illustrated as present in Figure 1. As seen in the diagram, the surface of Fe_3_O_4_ is coated with hydroxyl groups (─OH), and the surface interactions with l‐glu occur in the regions indicated by dashed lines in the diagram. These dashed lines represent weak interactions (hydrogen bonds and/or electrostatic interactions) rather than direct covalent bonding. It has been stated that Amp binding occurs via hydrogen bonds and electrostatic interactions on the glutamic acid–functionalized surface.

**Figure 1:**
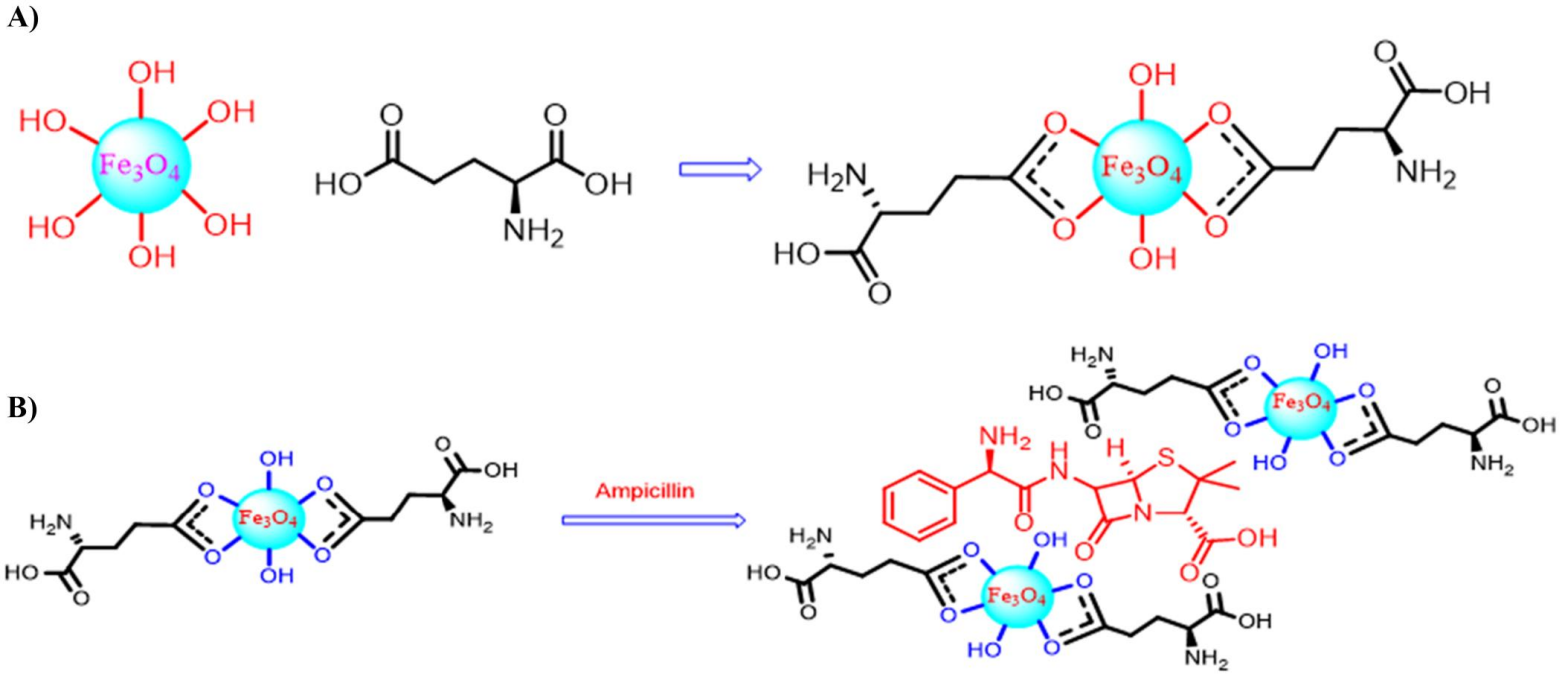
Schematic illustration of l‐glutamic acid functionalized Fe_3_O_4_ NPs (A) and l‐glu–Fe_3_O_4_–Amp NPs (B). NPs, nanoparticles.

Whether the surface modification of Fe_3_O_4_ NPs was achieved with l‐glu and whether the synthesis of l‐glu–Fe_3_O_4_–Amp took place was confirmed according to the FTIR, ZETAsizer, SEM based characterization results.

The functional groups of the samples were analyzed using FTIR spectroscopy in a range of 500–4000/cm (Figure 2). The strong characteristic peaks were observed at 3180, 2323, 2081, 1393, and 550/cm for Fe NPs alone, at 3330, and 1760, 1696, 1501, 1377, 1302, 725, and 639/cm for Amp alone, at 3032, 2080, 1637, 1509, 1308, and 1045/cm for Glu alone and at 3325, 1769, 1691, 1643, 1507 and 1377, 1306, 1212, and 591/cm for the formulation, respectively. The shifts in the ─OH stretching bands and in the amide I region, sensitive to C═O stretching and N─H vibrations, provide insight into the presence of hydrogen bonding within the formulation. Additionally, the 1150–1000/cm region was attributed to C─O, C─N stretching vibrations with the β‐lactam ring of Amp, which suggests interactions between Amp and the nanoformulation. The functional groups and chemical interactions in the nanoformulation were directly demonstrated by the FTIR spectra.

**Figure 2:**
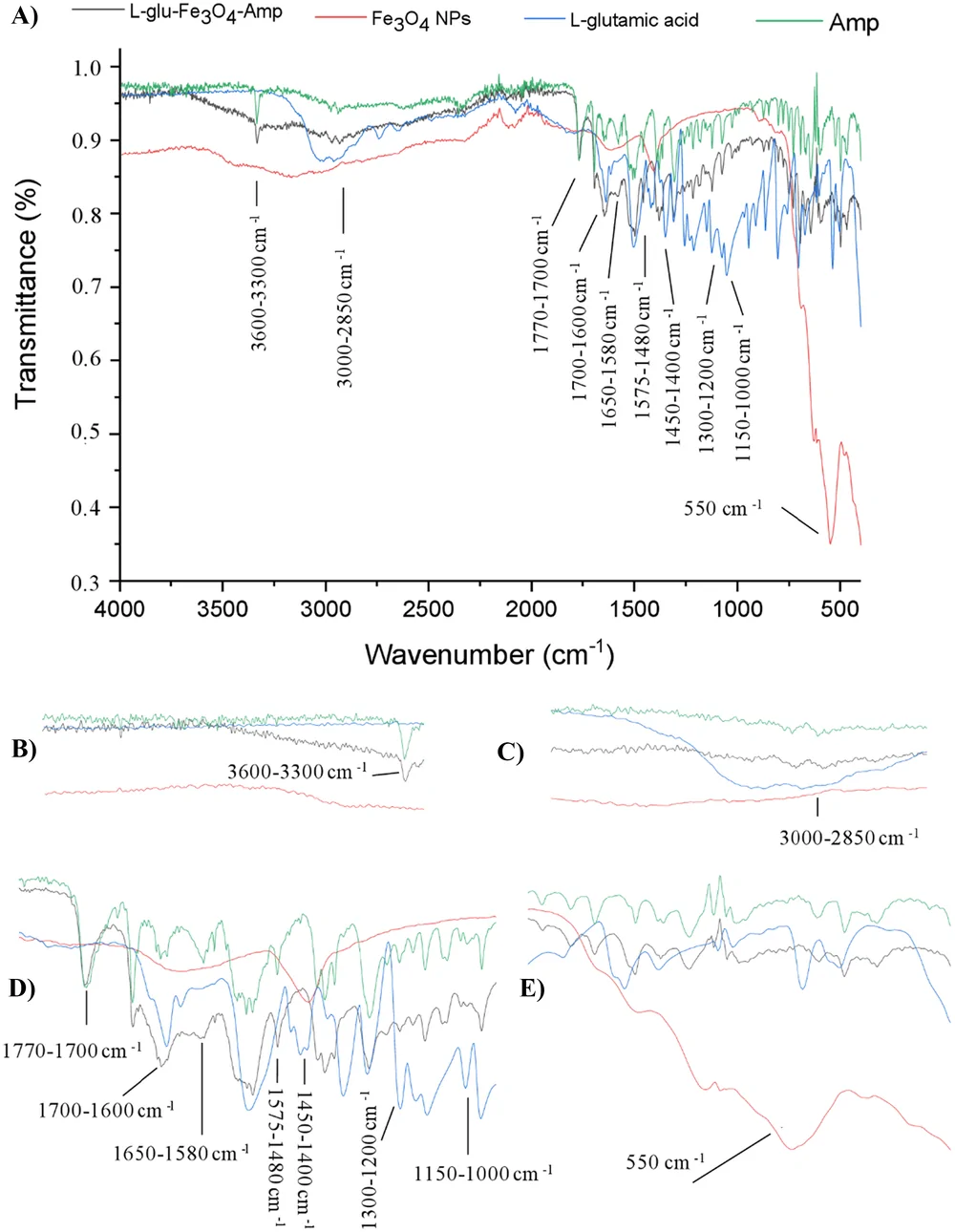
FTIR spectra results of l‐glu–Fe_3_O_4_–Amp (black), Fe_3_O_4_ NP (red), l‐glutamic acid (l‐glu) (blue), ampicillin (Amp) (green). The data are presented in the range of 4000–500/cm (A), 4000–3300/cm (B), 3300–2800/cm (C), 1900–1100/cm (D), and 800–400/cm (E). FTIR, Fourier transform infrared; NPs, nanoparticles.

SEM images revealed that the formulation exhibited strong agglomeration and had a spinel structure (Figure 3A). EDX spectroscopy demonstrated that the formulation contained C, N, O, S, and Fe elements (Figure 3B). On the basis of the ZETAsizer/potential assay, the size of l‐glu–Fe_3_O_4_–Amp was determined to be 164 nm (Figure 3C), PDI value was 0.68, and it has a negative charge value (−16.6 mV). Additionally, the size distribution was examined under different environmental conditions, such as pH 4 (Figure 4A, pH 10 (Figures 4B), 5% FBS (Figures 4C), and 10% FBS (Figure 4D). The sizes were determined as 606, 747, 146.2, and 174.3 nm, and have a negative charge value as −4.38, −6.05, −1.87, and −9.5 mV, respectively.

**Figure 3:**
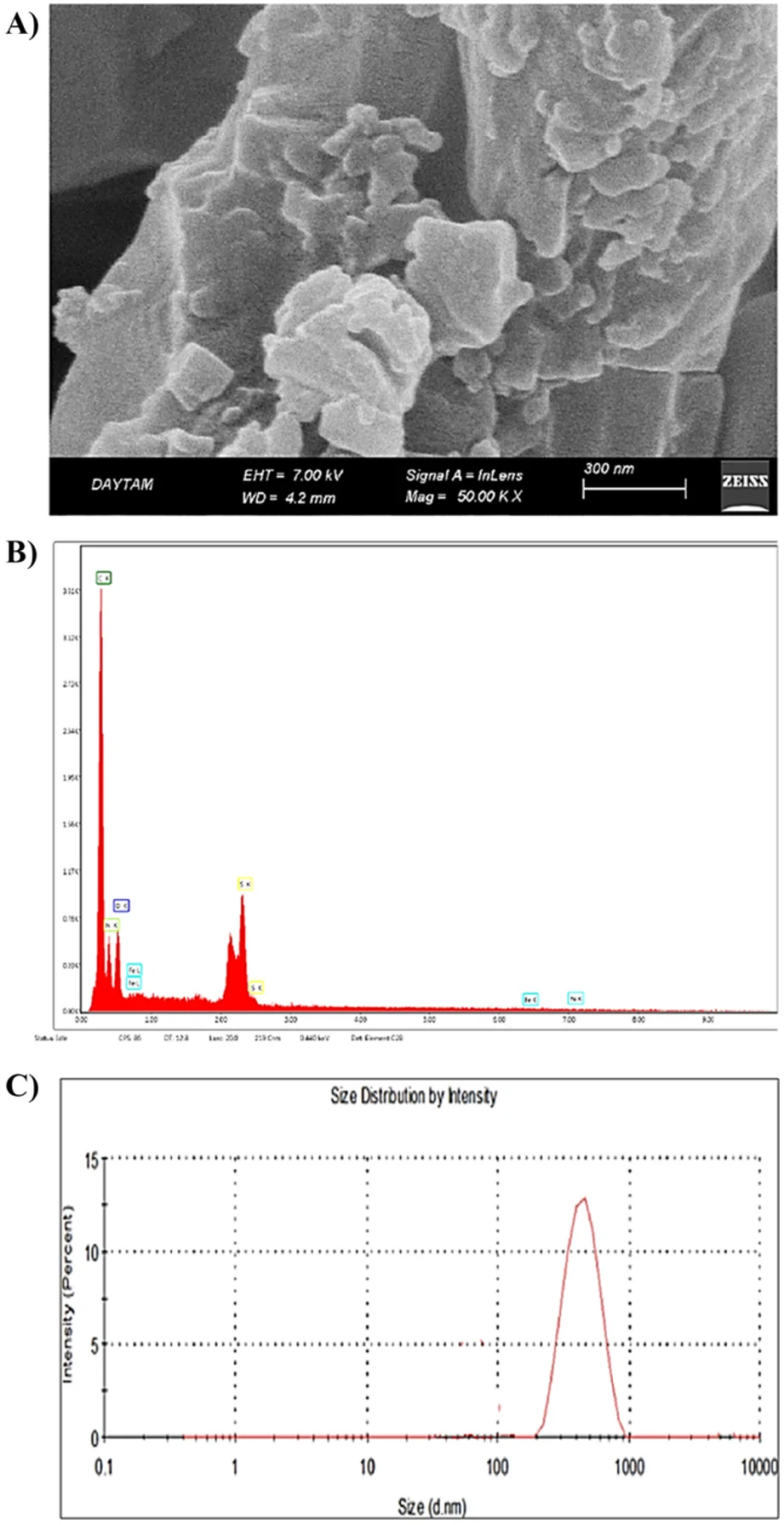
Scanning electron microscope images (A), X‐ray spectroscopy (EDX) (B), and size distribution of l‐glu–Fe_3_O_4_–Amp by ZETAsizer (C). Amp, ampicillin; EDX, energy‐dispersive X‐ray; l‐glu, l‐glutamic acid.

**Figure 4:**
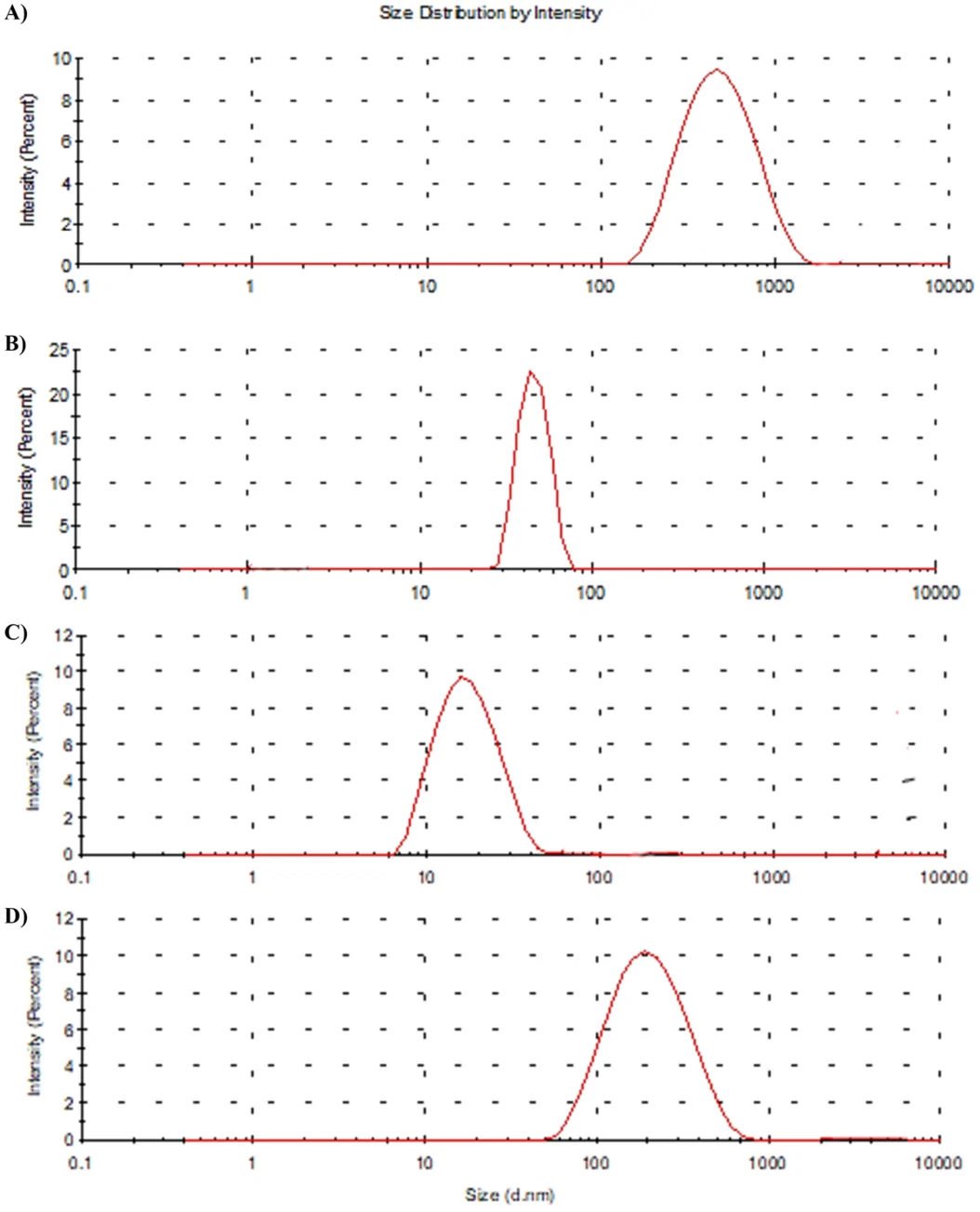
Size distribution of l‐glu–Fe_3_O_4_–Amp in different environmental conditions, as pH 4 (A), pH 10 (B), 5% FBS (C), and 10% FBS (D) by ZETAsizer. Amp, ampicillin; FBS, fetal bovine serum; l‐glu, l‐glutamic acid.

### 3.2 In Vitro Release Profile

A linear calibration curve was obtained by measuring the absorption of Amp concentrations as shown in Figure 5A. The release of Amp and l‐glu–Fe_3_O_4_–Amp was then studied in PBS (pH 7.4) at 37°C, and the concentration of released Amp was measured at different time points as presented in Figure 5. The Amp antibiotic was loaded onto l‐glu–Fe_3_O_4_, and the encapsulation and DL efficiencies were determined to be 99.8% and 18.71% (w/w), respectively, using UV–Vis spectroscopy. In vitro release of Amp from nanoformulation was studied at pH 7.5; the results are depicted in Figure 5B. The formulation demonstrated a prolonged‐release profile, with cumulative drug release increasing from nearly 13% at 0.5 h to 30% at 2 h, then transitioning to a slower phase that reached about 41% at 12 h. In comparison, free Amp exhibited a fast‐initial release, attaining nearly 10%–13% in the first hour and maintaining a virtually stable level thereafter. Approximately 30% of the drug was found to be released within 2 h. To study the release mechanism, three kinetic models were evaluated, and the correlation coefficient (*R* ^2^) of each model was used to assess the fit. The value of *R* ^2^ for all the models was high, as presented in Table 1. In the Korsmeyer–Peppas model, the *n* value was determined as 0.35271, which corresponds to a Fickian diffusion mechanism (0.45 ≤ *n*). This indicates that the nanoformulation can sustain therapeutic concentrations of the active drug for an extended duration, which is beneficial for enhancing antibacterial activity and minimizing dose frequency.

**Figure 5:**
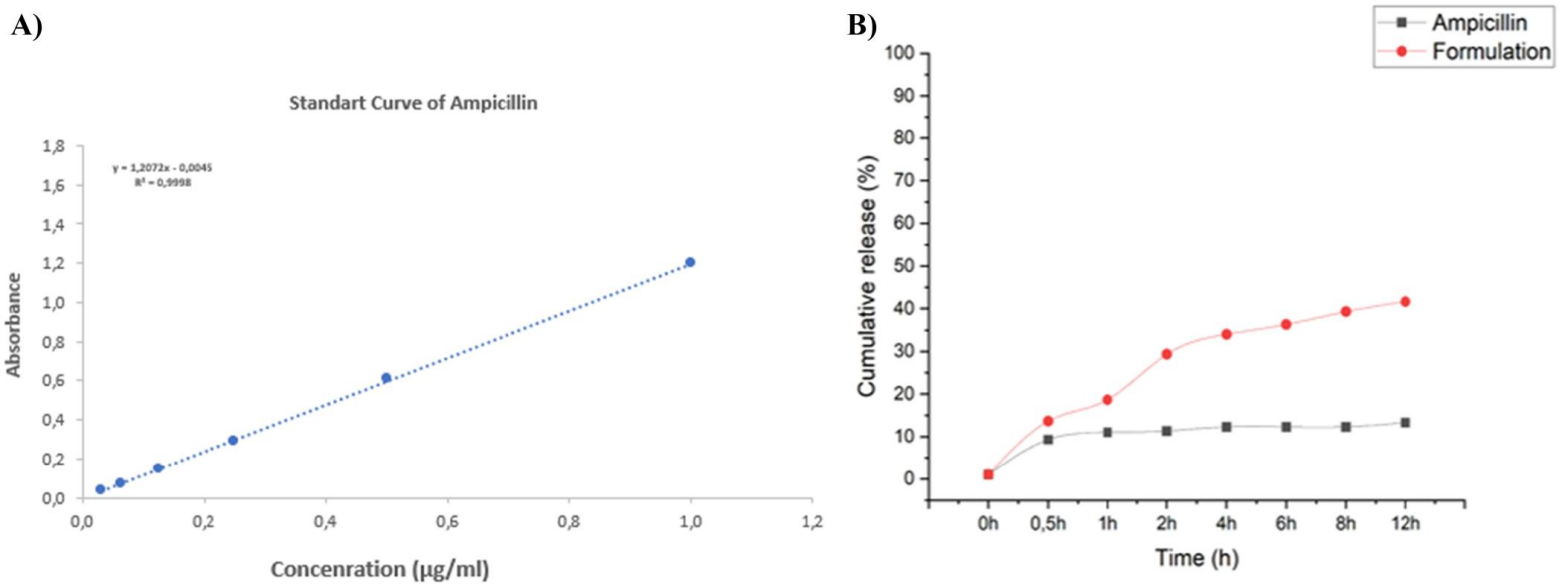
Standard curve graphic of ampicillin (Amp) (A), in vitro release profile of Amp (black), l‐glu–Fe_3_O_4_–Amp (red) (B). l‐glu, l‐glutamic acid.

**Table 1:** The tested kinetic models fitted to ampicillin release data.

	*R* ^2^	*K*
Zero‐order model	0.77111	2.2251
Higuchi model	0.91744	11.7726
Korsmeyer–Peppas model	0.94386	0.1693

### 3.3 Molecular Docking

Molecular docking analysis revealed that the l‐glu–Fe_3_O_4_–Amp ligand exhibits a high binding affinity toward the PBP1a protein (binding affinity: −9.3 kcal/mol), indicating a strong and stable ligand–protein interaction (Figure 6A). The calculated cavity volume of 1711 Å^3^ suggests the presence of sufficient steric space to accommodate the ligand, providing an accessible binding environment despite the NP formulation. Polar amino acid residues such as Ser, Thr, Lys, and Arg contribute to hydrogen bonding and electrostatic interactions, while aromatic residues, including Trp, Tyr, and Phe, provide hydrophobic stabilization. This amino acid interaction profile is consistent with the well‐established binding mechanisms of β‐lactam antibiotics to PBPs, indicating that Amp retains its target recognition capability even after conjugation to the NP. To further validate this observation, free Amp was docked to PBP1a under identical conditions as a control ligand. Free Amp exhibited a comparable binding affinity (−8.8 kcal/mol) and interacted with largely overlapping amino acid residues within the same binding cavity (Figure 6B). The slightly more favorable binding affinity observed for l‐glu–Fe_3_O_4_–Amp compared with free Amp suggests that nanoformulation did not hinder the binding of the Amp pharmacophore and may contribute to a modest stabilization of the ligand–protein complex. The binding affinity, cavity size, and contact residues for the ligand summarized in Table 2. The visual representation of the docking conformation and interaction sites provided in Figure 6.

**Figure 6:**
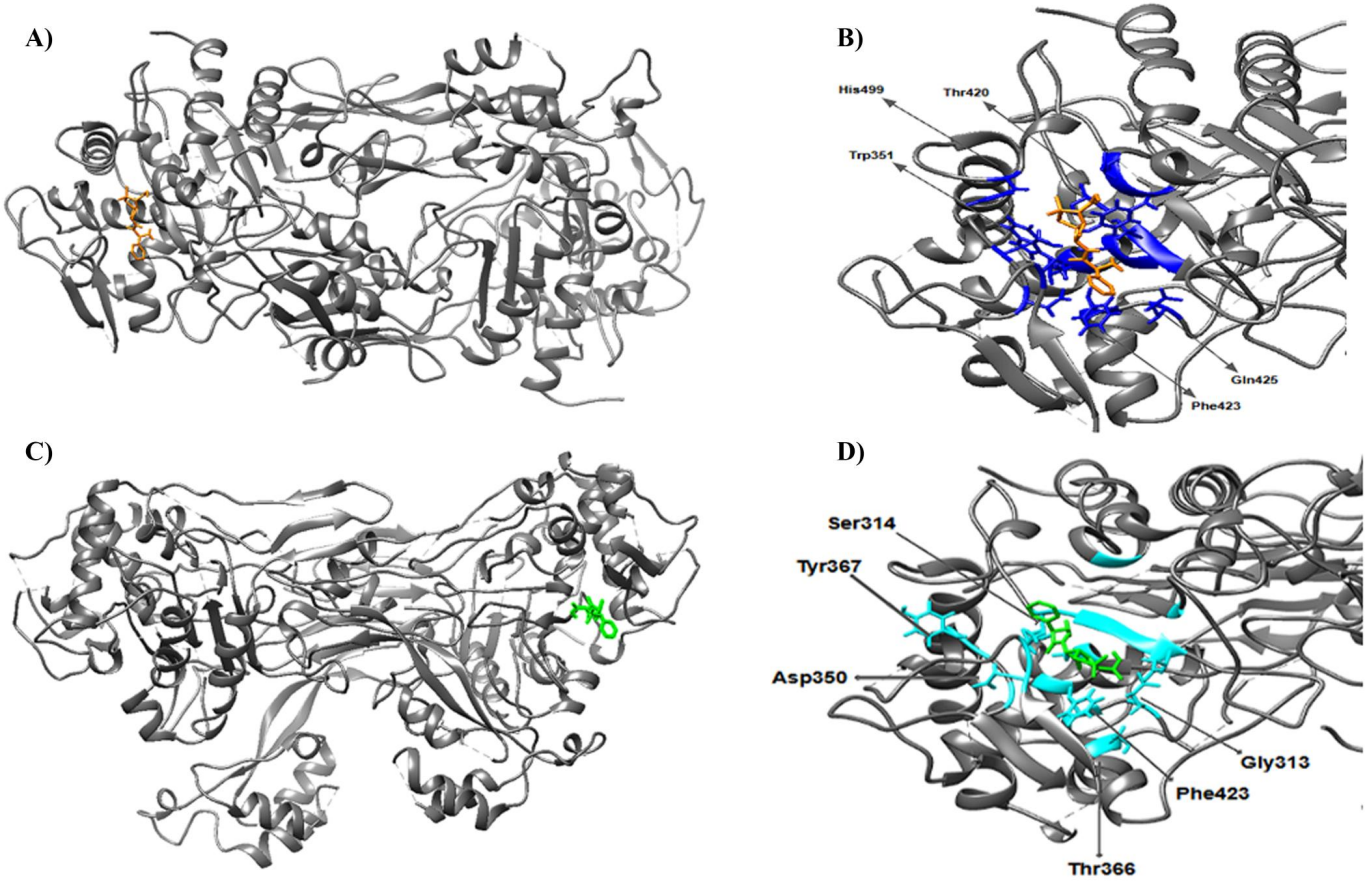
Molecular docking visualization of the predicted binding mode of free ampicillin (Amp) (A, B) and l‐glu–Fe_3_O_4_–Amp NPs (C, D) within the PBP1a binding pocket. Overall ribbon representation of the PBP1a–ligand docking complex showing the location of the docked ligand within the protein structure (A–C). Enlarged view of the predicted binding pocket highlighting the docked ligand and representative predicted binding‐pocket residues (B–D). l‐glu, l‐glutamic acid; NPs, nanoparticles; PBP, penicillin‐binding protein.

**Table 2:** Binding energy (docking score) and contact residues of l‐glu–Fe_3_O_4_–Amp ligand with PBP1a.

Receptor	Ligand	Vina docking score (kcal/mol)	Cavity volume (Å^3^)	Contact residues
PBP1a	l‐glu–Fe_3_O_4_–Amp	−9.3	1711	GLY313 SER314 LYS317 SER346 ARG347 ILE348 SER349 ASP350 TRP351 ASN352 ARG353 THR366 TYR367 SER368 SER369 ASN370 LEU416 THR420 PHE423 GLN425 HIS499 ALA500 LYS513 THR514 GLY515 THR516 ALA517 GLN518 TYR527 TYR534 TYR566 GLU567
PBP1a	Ampicillin	−8.8	1711	GLY313 SER314 LYS317 SER346 ARG347 ILE348 SER349 ASP350 TRP351 THR366 TYR367 SER368 SER369 ASN370 LEU416 THR420 PHE423 GLN425 VAL492 HIS499 ALA500 LYS513 THR514 GLY515 THR516 ALA517 GLN518 TYR527 TYR534 TYR566 GLU567

Abbreviations: Amp, ampicillin; l‐glu, l‐glutamic acid; PBP, penicillin‐binding protein.

### 3.4 Agar Well Diffusion Assay

Free Fe_3_O_4_ NPs and l‐glu–Fe_3_O_4_ NPs did not show any antimicrobial activity against both bacterial strains (Figure 7A,C). Both Amp and the formulation (l‐glu–Fe_3_O_4_–Amp) were determined to display antimicrobial efficacy toward *S. aureus* and *B. cereus*. At Amp concentrations of 1, 10, and 100 µg, the inhibition zones were found to be 11, 25, and 32 mm against *S. aureus*, and 23, 33, and 35 mm against *B. cereus*, respectively. The l‐glu–Fe_3_O_4_–Amp nano formulation resulted in inhibition zones of 8, 14, and 27 mm against *S. aureus*, and 20, 26, and 34 mm against *B. cereus* when used at concentrations of 1, 10, and 100 µg, respectively (Figure 7B,D). The graphical representation of the inhibition zones was presented in Figure 8A,B.

**Figure 7:**
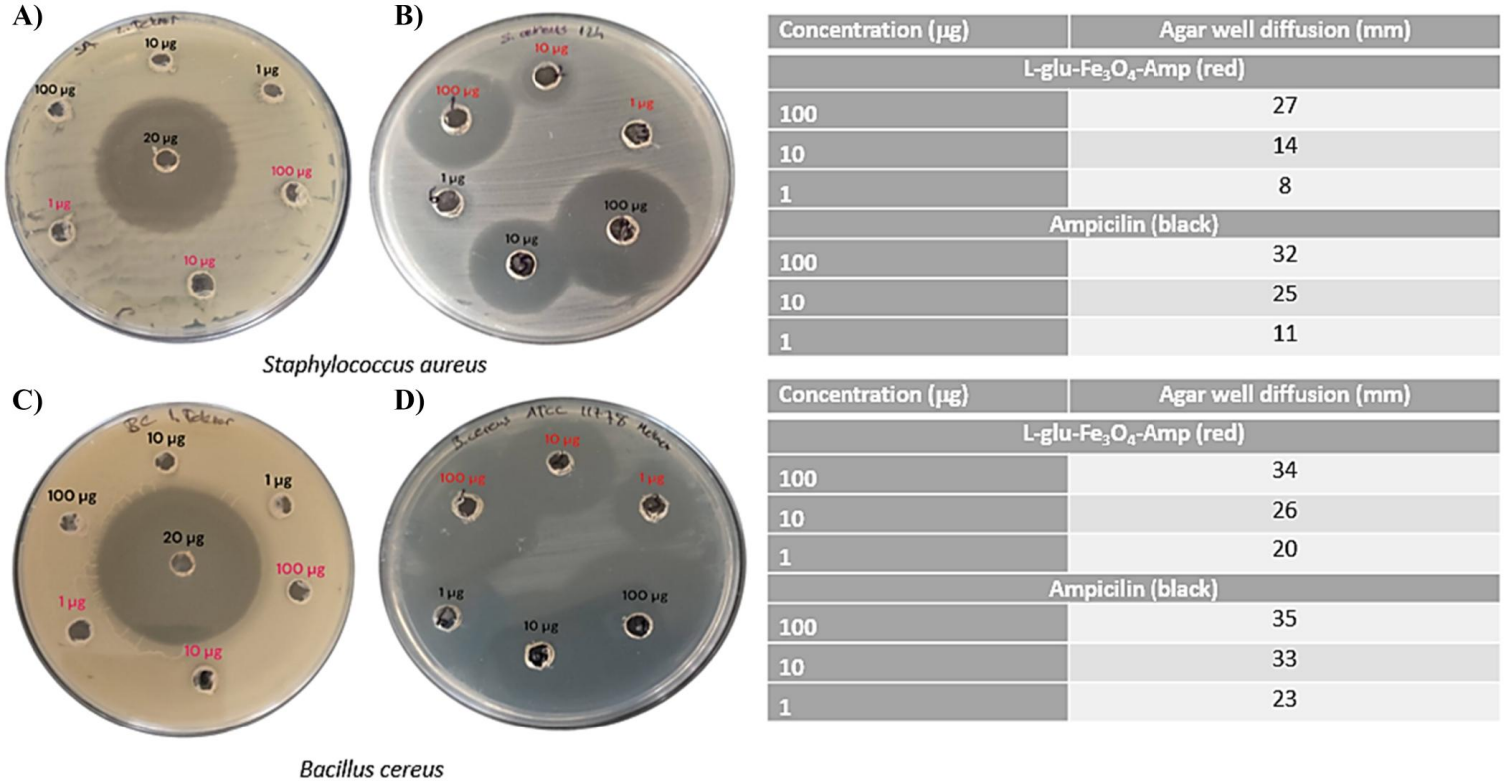
Antimicrobial activity results at different concentrations of free Fe_3_O_4_ NPs, l‐glu–Fe_3_O_4_ NP, l‐glu–Fe_3_O_4_–Amp NP, and ampicillin (Amp) against *Staphylococcus aureus* (A, B), free Fe_3_O_4_ NPs, l‐glu–Fe_3_O_4_ NP, l‐glu–Fe_3_O_4_–Amp, and Amp against *Bacillus cereus* (C, D). l‐glu, l‐glutamic acid; NPs, nanoparticles.

**Figure 8:**
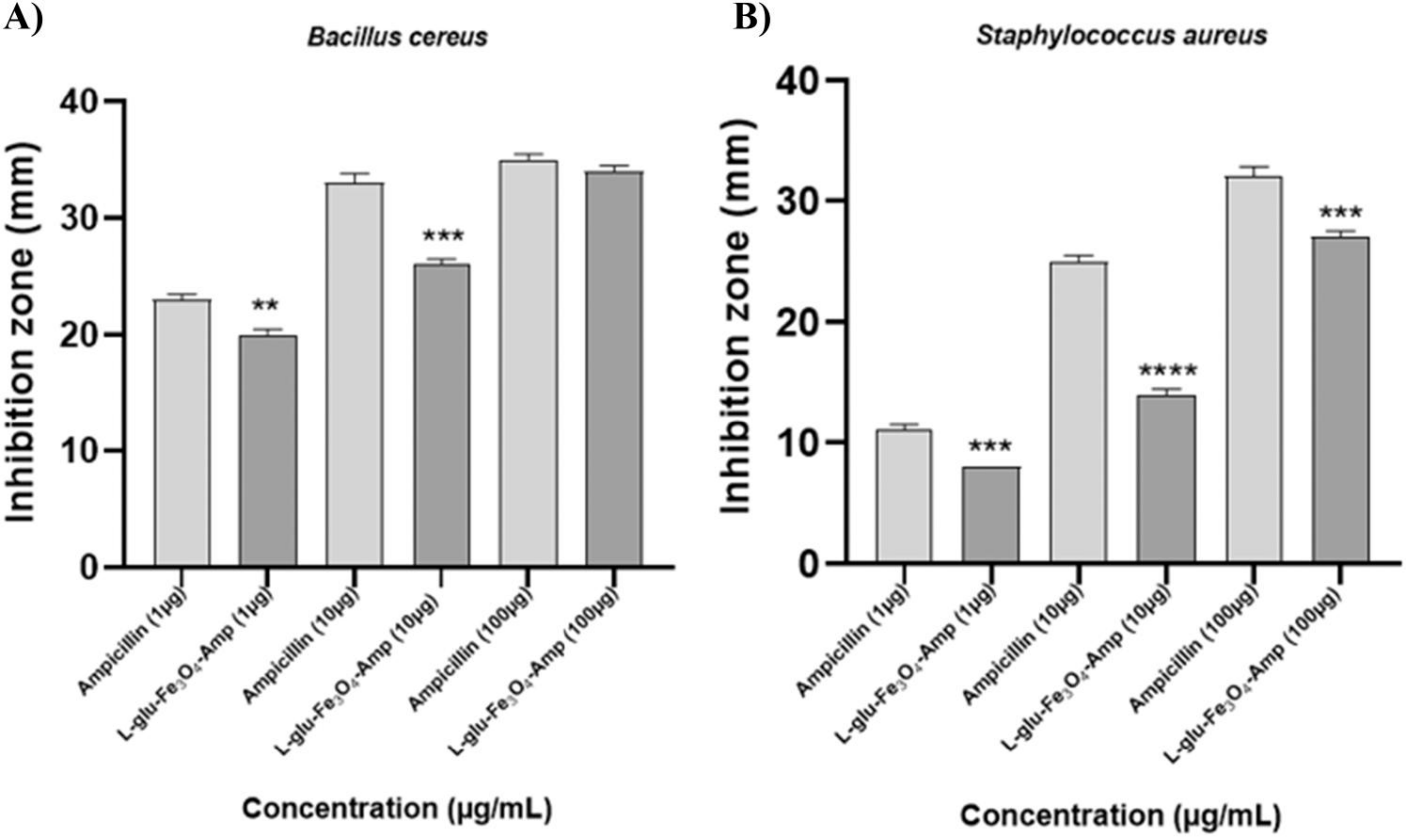
The inhibition zone of ampicillin (Amp) and l‐glu–Fe_3_O_4_–Amp at different concentrations against *Bacillus cereus* (A) and *Staphylococcus aureus* (B) (*n* = 3). Statistically significant differences in applied concentrations (110,100 µg/mL) were presented for *B. cereus* as **p < 0.0015 and ***p < 0.0002, for *S. aureus* as ***p < 0.0004, ****p < 0.0001, and ***p < 0.0008, respectively. l‐glu, l‐glutamic acid.

### 3.5 Minimum Inhibitory Concentration

The MIC values reflect the ability of Amp and l‐glu–Fe_3_O_4_–Amp to inhibit the growth of *B. cereus* and *S. aureus* strains (Table 3). MIC values of Amp and l‐glu–Fe_3_O_4_–Amp against *S. aureus* were lower than the values measured against *B. cereus*. The results summarized in Table 2 also indicate that against *S. aureus*, the MIC value of Amp (7.8 µg/mL) was lower than that of l‐glu–Fe_3_O_4_–Amp (15.6 µg/mL). The MIC values of Amp and l‐glu–Fe_3_O_4_–Amp against *B. cereus* were 62.5 and 31.2 µg/mL. The next stages of the study were designed according to the obtained MIC values.

**Table 3:** Minimum inhibitory concentration of Amp and l‐glu–Fe_3_O_4_–Amp against the bacterial strains.^a^

Bacterial strain	Amp (μg/mL)	l‐glu–Fe_3_O_4_–Amp (μg/mL)
*Bacillus cereus* (ATCC 157717)	62.5 ± 02	31.2 ± 01
*Staphylococcus aureus* (ATCC 25923)	7.8 ± 02	15.6 ± 02

Abbreviations: Amp, ampicillin; CFU, colony‐forming units; l‐glu, l‐glutamic acid.

^a^ The experiment was done in triplicate; the bacterial count was 108 CFU/mL.

### 3.6 Growth Kinetic Analysis

In the further stages of study, MIC values of l‐glu–Fe_3_O_4_–Amp against *B. cereus* and *S. aureus* (respectively, 31.2 ± 01 and 15.6 ± 02 µg/mL) were selected, while Amp‐based experiments were conducted at its commercial concentration of 20 µg/mL. Growth kinetic analysis was performed both in the presence of test materials. The test material‐free medium was used as a negative control. *B. cereus* (Figure 9A) and *S. aureus* (Figure 9B) were treated with different doses of test materials and then incubated at 37°C at a shaking speed of 120 rpm. OD_600 nm_ was monitored for 10 h.

**Figure 9:**
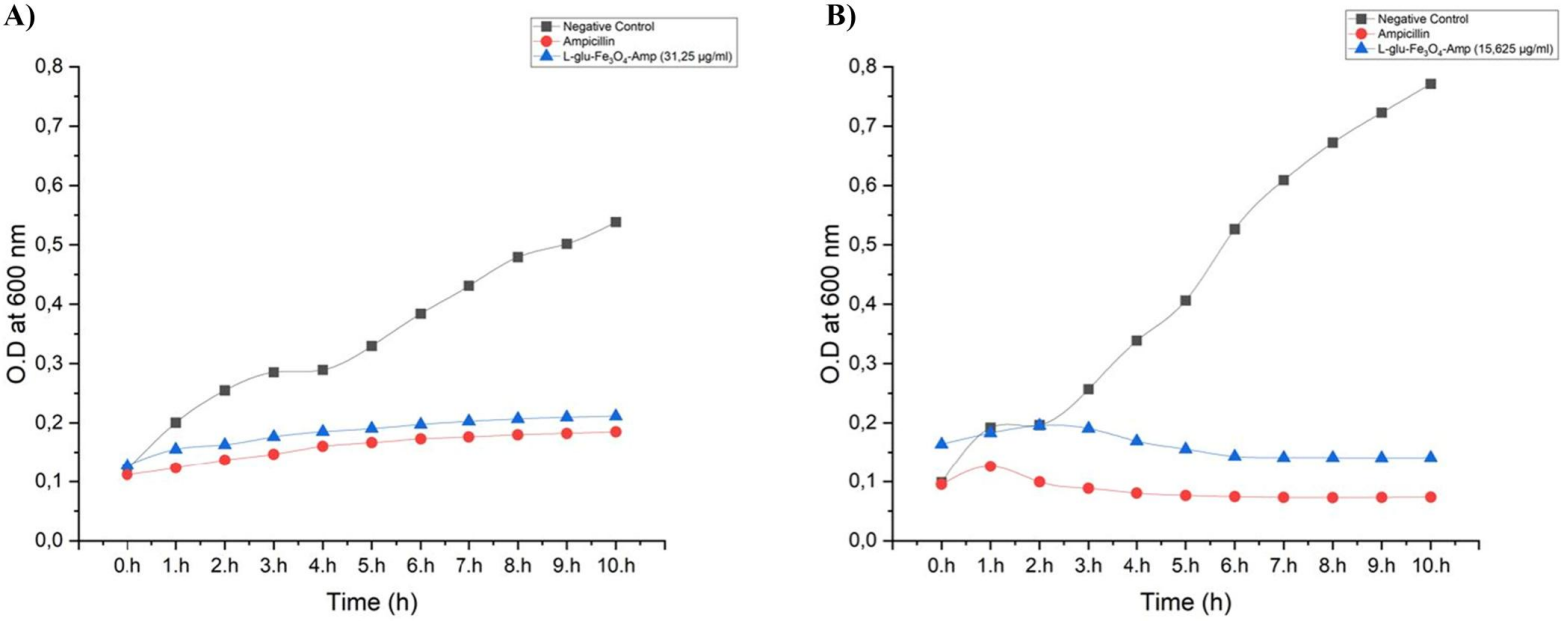
Growth kinetics results of *Bacillus cereus* (A) and *Staphylococcus aureus* (B) in the presence and absence of l‐glu–Fe_3_O_4_–Amp at the log phase of growth kinetics. Triplicate experiments were performed for each reaction (*n* = 3). Amp, ampicillin; l‐glu, l‐glutamic acid; OD, optical density.

The cell densities of the untreated groups of both bacteria were found to increase continuously over time. On the contrary, Amp alone or l‐glu–Fe_3_O_4_–Amp alone (treatment groups) was ascertained to affect negatively cell growth in both bacteria (Figure 9). The cell density of *B. cereus* showed a very small increase in all treatment groups until 6 h; however, it did not show a significant increase in any of the groups after 6 h and remained constant until 10 h (Figure 9A). In *S. aureus*, no cell growth was detected in any treatment group during the 10 h incubation period (Figure 9B). In short, the results revealed that none of the bacteria were capable of surviving in the presence of Amp or the formulation.

### 3.7 Measurement of Oxidative Stress (ROS) Level

After the treatment with l‐glu–Fe_3_O_4_–Amp (at 15.6 and 31.2 µg/mL for *S. aureus* and *B. cereus*, respectively) or Amp (at a commercial concentration of 20 µg/mL), the ROS formation level in bacterial cells was analyzed using the DCFH‐DA fluorescence method, and the results were presented in Figure 10. The measurement was also performed in culture media in the absence of any treatment, indicating the natural production of ROS. According to results, the presence of both test samples resulted in a significant increase in the fluorescence intensity, which is relatively correlated with the higher amount of ROS production for both *B. cereus* (Figure 10A) and *S. aureus* (Figure 10B) cells. In addition, the results manifested that the amount of ROS produced is higher in l‐glu–Fe_3_O_4_–Amp group as compared with the Amp group.

**Figure 10:**
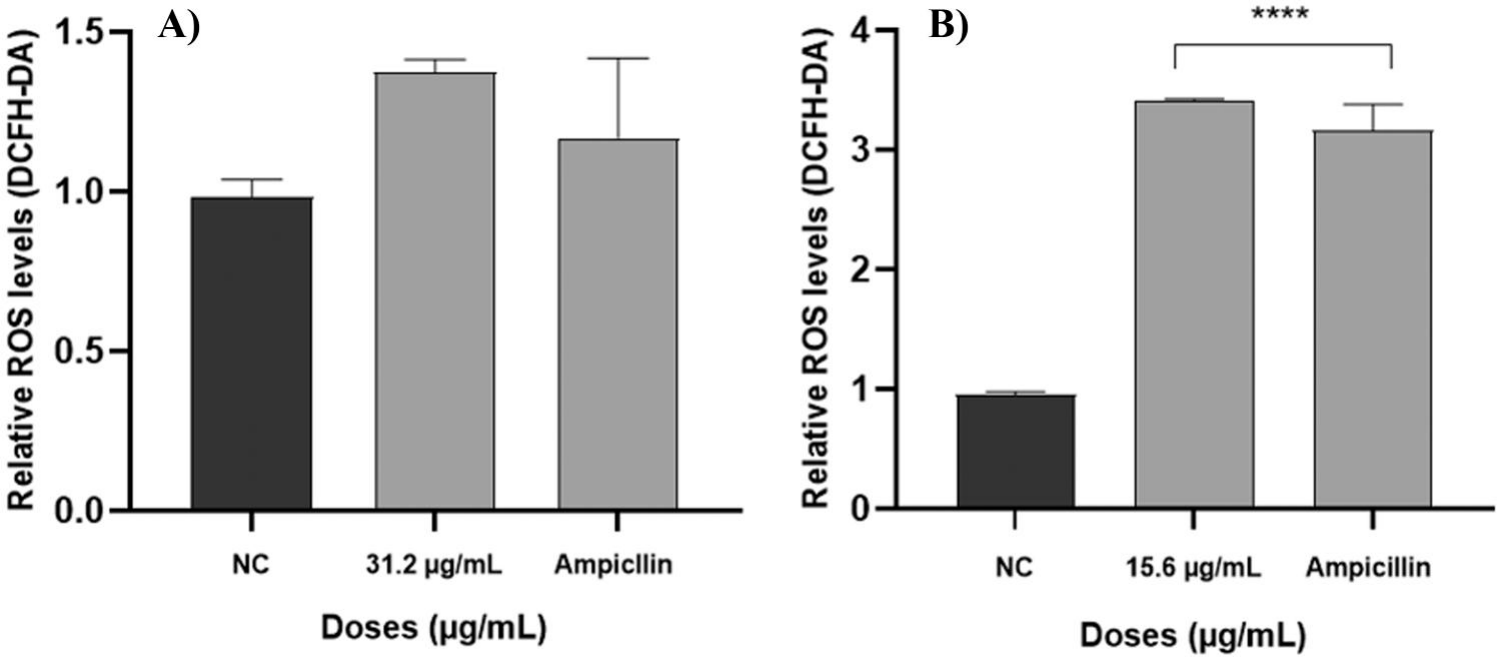
Effects of l‐glu–Fe_3_O_4_–Amp and ampicillin (Amp) on intracellular ROS level on *Bacillus cereus* (A) and *Staphylococcus aureus* (B) after 4 h incubation, and cells were stained with 20 µM DCFH‐DA. NC: negative control (not treated bacterial cells). Data represent the mean fluorescence signal with ±SD (*n* = 3), with calculation against the negative controls, and at least 10.000 events were collected per sample. Statistical analysis using one‐way analysis of variance followed by Duncan's multiple comparison test confirmed that the differences were significant compared with the untreated control group (*p* < 0.001). DCFH, 2′,7‐dichlorofluorescein; l‐glu, l‐glutamic acid; ROS, reactive oxygen species.

### 3.8 LIVE/DEAD BacLight Fluorescence Test

The LIVE/DEAD BacLight fluorescence kit emits green fluorescence in the presence of viable cells, since SYTO9 stains the intact membranes of viable cells, resulting in green emission. PI stains cells with compromised membranes, and its emission wavelength is in the red. The antibacterial efficacy of both the drug and the nanoantibiotic, as determined by the interaction pattern, is further investigated using the LIVE/DEAD BacLight fluorescence kit on a flow cytometer. As illustrated in Figure 11A, the negative control (untreated bacterial cells) exhibited green fluorescence, indicating a cell viability of 74.35%. The nonviable cell number of *B. cereus* was determined to be in 17.48% and 18.40% for Amp and l‐glu–Fe_3_O_4_–Amp groups, respectively. Figure 11B depicts that untreated cells (negative control) of *S. aureus* had a green fluorescence, inferring a cell viability of 73.97%. In the Amp and l‐glu–Fe_3_O_4_–Amp groups of *S. aureus*, nonviable cell ratios of 52.05% and 51.66%, were measured, respectively.

**Figure 11:**
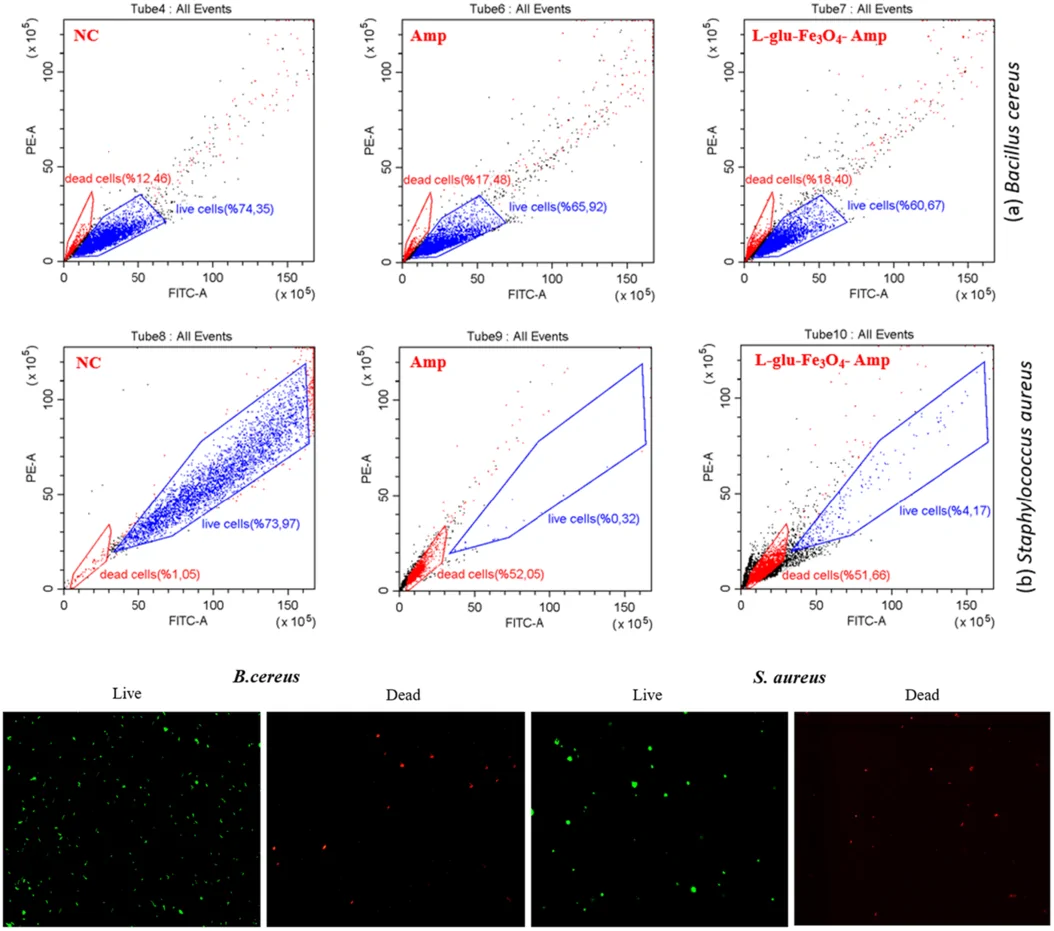
Flow cytometry analysis of cell viability on *Bacillus cereus* (A) and *Staphylococcus aureus* (B) by staining with propidium iodide and Cyto 9 on the *X*‐ and *Y*‐axes. Populations are colored according to viable cells in green and nonviable cells in red. Fluorescence microscopic images of live and dead cells on *B. cereus* and *S. aureus* are presented. Amp, ampicillin; l‐glu, l‐glutamic acid; NC, negative control.

### 3.9 Cytotoxicity Assay

The cell toxicities of l‐glu–Fe_3_O_4_–Amp (Figure 12A) and Amp (Figure 12B) were tested on L929 cell line by utilizing WST‐1‐based toxicity test, which is widely used to evaluate the biocompatibility of materials. The data elucidated that when compared with the control group, all concentrations of AMP tested from 6.25 to 500 µg/mL reduced the cell viability in a concentration‐dependent manner. Especially, Amp was found to decrease statistically cell viability in concentration range from 12.5 to 500 µg/mL (Figure 12B). On the contrary, as seen from Figure 12A, none of the tested concentrations of l‐glu–Fe_3_O_4_–Amp reduced the viability of the L929 cell line. This finding shows that the formulation does not cause side effects in healthy cells and is an advantage for their safe use.

**Figure 12:**
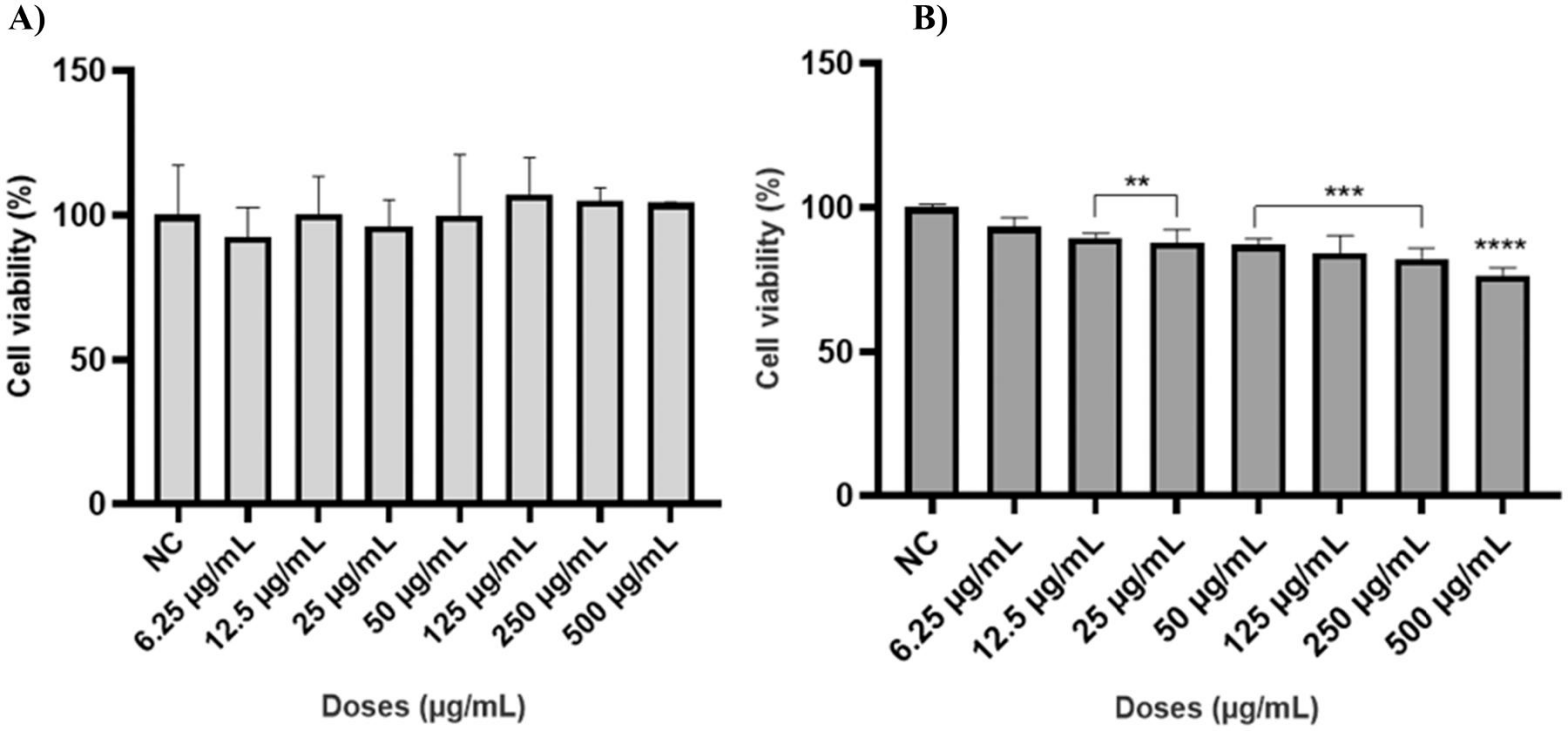
Cell viability results after 24 h exposure of l‐glu–Fe_3_O_4_–Amp (A) and Amp (B) at different concentrations in L929 cell lines by using WST‐1 assay. Data are presented as mean ± SD (*n* = 3) from three independent replicate experiments. The * indicates differences between treatment groups compared with the negative control group (NC). *p* < 0.01; *p* < 0.001; *p* < 0.0001. Amp, ampicillin; l‐glu, l‐glutamic acid; WST‐1, Water‐Soluble Tetrazolium 1.

### 3.10 TEM Imaging

Using TEM to investigate uptake and NP–cell interactions in bacteria can also allow observation of morphological changes. This technique leads to the identification of individual particle uptake as well as their interaction because TEM can image distinctive membrane characteristics.

In this study, TEM analysis revealed that l‐glu–Fe_3_O_4_–Amp NPs adhered to the surface of bacterial membranes, disrupted the cell membrane integrity, and caused electron‐dense regions (Figure 13A,B). Untreated cells served as control group (Figure 13C,D). For example, protrusions on the membrane at sites where the formulation binds indicate membrane damage or disruption of its integrity.

**Figure 13:**
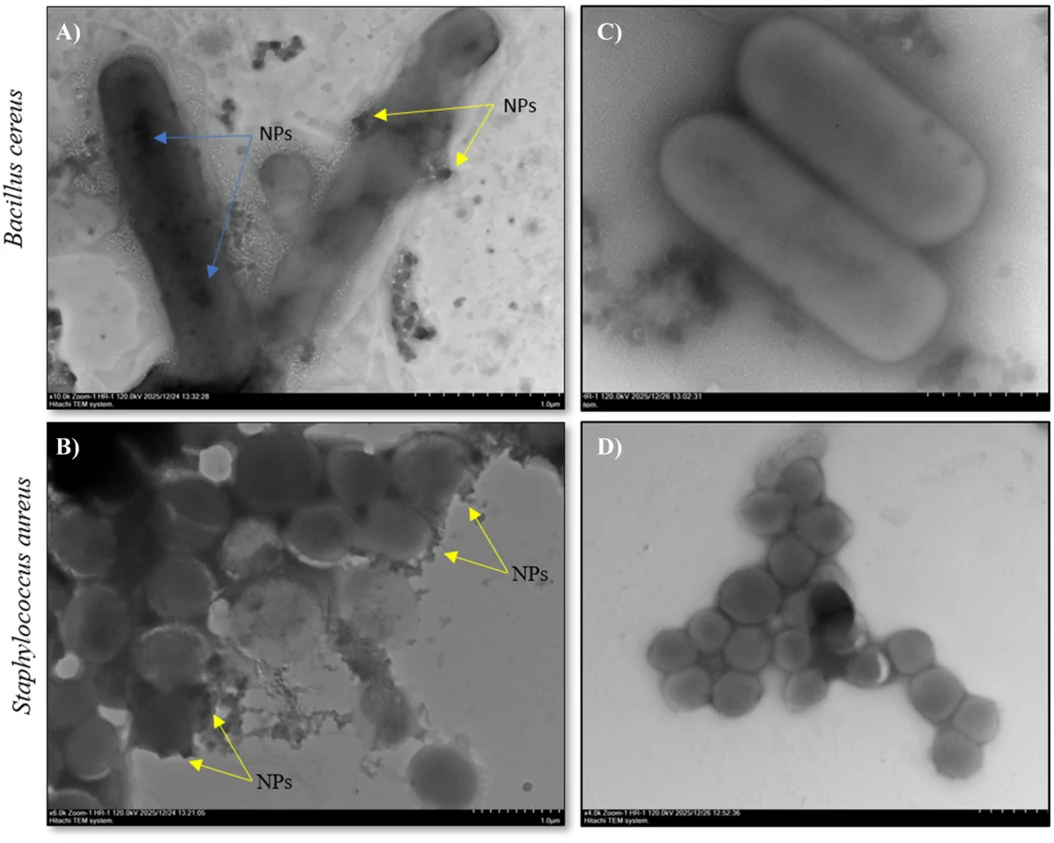
TEM images of *Bacillus cereus* (A) and *Staphylococcus aureus* (B) treated with l‐glu–Fe_3_O_4_–Amp and untreated cells served as a negative control (C, D). Yellow arrows indicate membrane‐associated nanoparticles (NPs) and disruptions in membrane structures. The blue arrows represent NPs and electron‐dense regions within the cell. Amp, ampicillin; l‐glu, l‐glutamic acid; TEM, transmission electron microscopy.

### 3.11 Cellular Internalization in Bacteria

The nanoformulation was labeled with Oregon Green 488 fluorescent dye, and its interaction with bacterial cells was evaluated using confocal microscopy. The results were presented comparatively between untreated and treated bacterial cells, demonstrating that the fluorescent signal was highly localized to bacteria of both *B. cereus* and *S. aureus* (Figure 14). The uniform distribution of the fluorescent signal throughout the cell body, rather than just on the cell surface, implies that the nanodye is localized by both cellular internalization and surface adsorption.

**Figure 14:**
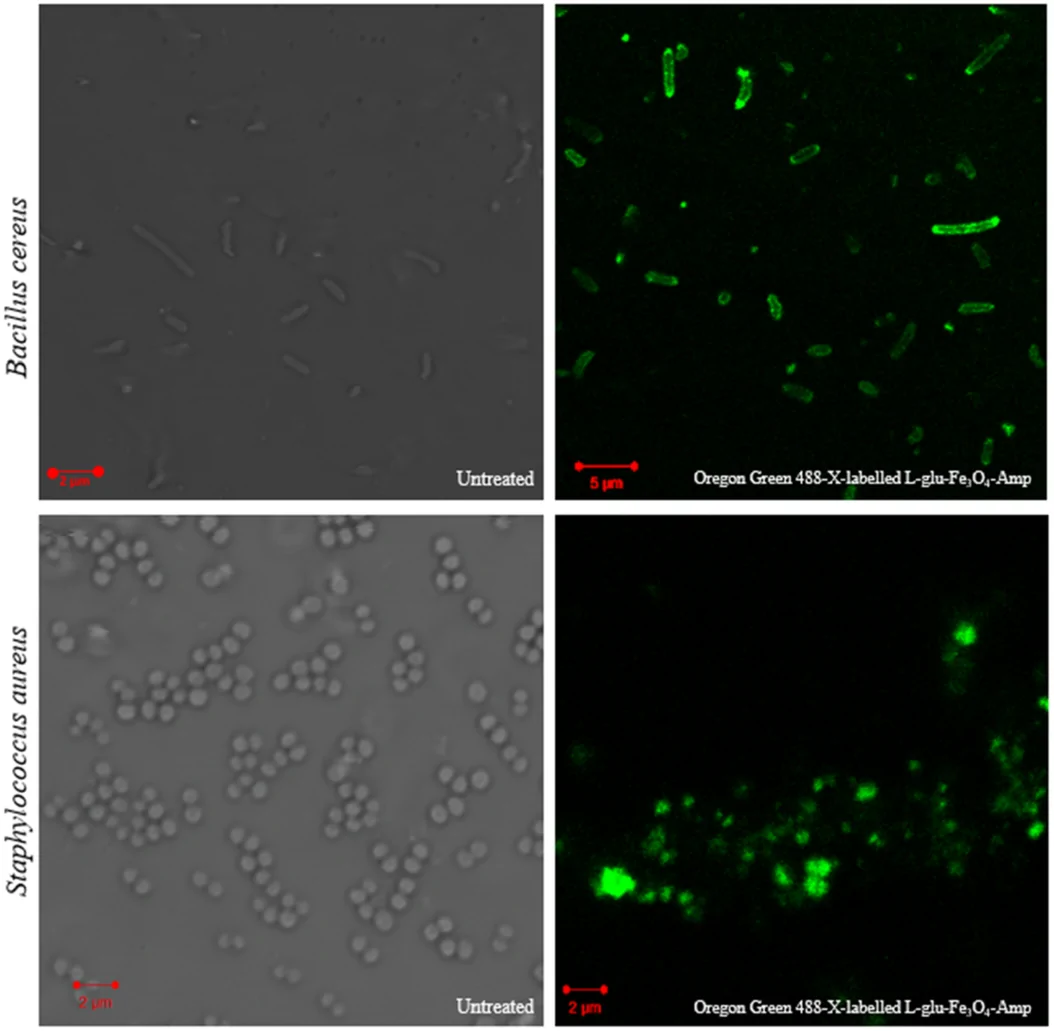
Confocal microscopy images in *Bacillus cereus* and *Staphylococcus aureus* with untreated and treated Oregon Green 488‐X‐labeled l‐glu–Fe_3_O_4_–Amp. Amp, ampicillin; l‐glu, l‐glutamic acid.

## 4 Discussion

Nanocarriers can reduce adverse impacts by enhancing the solubility and stability of antibacterial drugs. NPs‐based antimicrobial drug delivery systems are known to provide promising results in overcoming the resistance mechanisms developed by many pathogenic bacteria (Taubes 2008). Pothineni and Keller (2023) show that glycopeptide antibiotics such as vancomycin can be re‐effective against resistant bacterial strains by incorporating them into nanoparticular systems (Pothineni and Keller 2023). Sherafati Chaleshtori et al. (2024) demonstrated that controlled release of vancomycin with gelatin NPs enhanced infection control and bone repair in MRSA‐induced osteomyelitis models (Sherafati Chaleshtori et al. 2024).

Magnetic NPs are extensively utilized in biomedical sciences because of their biocompatibility, chemical stability, and magnetic properties (Mahdavi et al. 2013). In particular, magnetic NPs are utilized for targeted drug delivery via an external magnetic field, thereby enhancing the stability of pharmaceuticals against enzymatic or metabolic degradation. In a study conducted by Zhao et al. (2004), dextran‐coated superparamagnetic Fe_3_O_4_ NPs were aggregated via Con‐A treatment or functionalized with Con‐A‐conjugated nanosensors, and these NPs were capable of evaluating microbial metabolic activity and determining antimicrobial susceptibility in blood through the fast quantification of polysaccharides (Zhao et al. 2004). A recent study (U Din et al. 2024) proved the antibacterial and antioxidant properties of AgNPs coupled with the levofloxacin and ciprofloxacin antibiotics, alongside biologically produced NPs derived from *Moringa oleifera* and *Curcuma longa*. In a different study (Basu et al. 2004), supermagnetic iron oxide nanoprobes significantly facilitated the identification of *Mycobacterium avium* spp. *paratuberculosis* (MAP) and enabled the rapid quantification of MAP in milk and blood with excellent sensitivity. In a study, magnetic NPs were conjugated with vancomycin via dopamine, demonstrating potent inhibitory effects against both Gram‐positive and Gram‐negative bacteria and the ability to be directed to the target upon application of a magnetic field (Abdelaziz et al. 2022).

The widespread use of magnetic NPs includes (i) their ability to be directed to the infection site with the help of an externally applied magnetic field, (ii) the antibiotic is delivered directly to the target in infections in deep tissues, thus reducing systemic toxicity. This study focused on the design and synthesis of a nanoantibiotic delivery system consisting of Fe_3_O_4_ NPs, Amp, and l‐Glu as functional components.

It is well known that surface modifications are very important for the development of any nano‐based drug formulations. Most specific coating agents are reported to be toxic in the literature. For instance, cetyltrimethylammonium bromide (CTAB), a widely used cationic surfactant in NP synthesis, has been consistently reported to exhibit significant cytotoxicity (Wan 2015). Also, in the study of Zhang et al. (2015), the authors tested eleven commonly used surface coating agents for cytotoxicity, and they observed that six of the surface coating agents are cytotoxic (Zhang et al. 2015). The presence of l‐glu in the structure allows more drug to be loaded due to its amine groups being accessible for interaction with the hydroxide functional group on the surface of Fe_3_O_4_ NPs with reduce toxicity. Therefore, in the first stage of the study, l‐glu was employed to achieve the surface modification of Fe_3_O_4_ NPs. In the second stage, Amp was bound to the Fe_3_O_4_ NPs‐Glu to prepare the final formulation (l‐glu–Fe_3_O_4_–Amp NPs).

In the FTIR spectra, Fe NPs are known to exhibit the most prominent peaks at 400–700/cm. In this study, the strongest peaks were observed at 550 and 591/cm for Fe NPs alone and the formulation, respectively. Amide bands (I, II, and III) indicate the existence of proteins, peptides, or amino acids in a sample. In FTIR spectra, the absorbances peaks in the range of 1600 to 1700/cm are related Amid I and may be referred to C═O and C─N groups stretching vibration. Absorbances between 1480 and 1575/cm belong to Amide II and are explained by N─H bending and C─N stretching vibrations. Absorbances between 1200 and 1350/cm are assigned to Amide III (Hashim et al. 2010; Dawood et al. 2020; Ji et al. 2020). In this study, the characteristic amide I, amide II, and amide III peaks of l‐glu alone were around 1637, 1509, and 1308, respectively, as well as COO^−^ stretching vibrations around ~1600 and ~1400/cm. The formulation displayed amide I, amide II, and amide III bands at 1643, 1507, and 1306/cm, respectively. It is known that the Amp gives characteristic peaks at 3200–3600/cm (O─H stretching and N─H stretching). The β‐lactam carbonyl stretching of Amp was assigned to the band appearing at 1760–1780 and 1760–1790/cm (C═O stretching). In this study, the characteristic peaks for both Amp (green line) and the final formulation (black line) were detected at 3330 and 1769/cm. These specific bands in l‐glu alone, Fe NPs alone, and Amp alone were also observed in the final nanoformulation, proving that the final formulation was successfully prepared. The shifts and changes in these distinctive bands in the l‐glu–Fe_3_O_4_–Amp nanoformulation relative to its constituent parts indicate effective surface modification and interaction between components. The suggested interaction mechanisms, especially those involving hydrogen bonding and electrostatic interactions, are supported by these spectral shifts. EDX results showed that the formulation contained C, N, O, S, and Fe. It is known that Amp is made up of C, H, O, N, and S, while the chemical structure of l‐glu contains C, H, O, and N elements. On the basis of these results, we interpreted that S in the formulation was sourced from Amp, while N can be derived from Amp and/or l‐Glu. In addition, it was concluded that Fe in the formulation was derived from Fe_3_O_4_ NPs. This result confirmed that Fe_3_O_4_ NPs were incorporated into the formulation's structure. The physicochemical parameters of the formulation align with trends commonly documented in the literature for NP systems in physiologically relevant conditions. The relatively high polydispersity index (PDI = 0.68) indicates a broad size distribution, which has been frequently associated with heterogeneous dispersions and an increased tendency toward aggregation. The observed size increase under different pH conditions (~606 nm at pH 4 and ~747 nm at pH 10) indicates that the nanoformulation is pH‐sensitive, and this is consistent with the decrease in surface charge (−4.38 and −6.05 mV, respectively). It is noteworthy that in serum‐containing media (5% and 10% FBS), the particle sizes were measured as approximately 146 and 174 nm, respectively. The fact that the size is close to or even slightly smaller than the initial value, especially in 5% FBS, suggests that protein corona formation may play a stabilizing role on the particle surface. However, the significant decrease in zeta potential to low values (−1.87 and −9.5 mV) suggests that electrostatic stability may be masked by protein adsorption and that stability is likely achieved through steric interactions. The increase in size at 10% FBS can be explained by more intense protein interactions or partial aggregation. Additionally, the inclusion of Amp in the formulation may influence the observed behavior. Amp is a hydrophilic β‐lactam antibiotic featuring ionizable functional groups that can engage with the particle surface and the surrounding media. Such interactions may diminish the effective surface charge, hence contributing to the comparatively low zeta potential reported.

The primary criterion for controlled‐release systems is to sustain a desired drug concentration at the site of action. The controlled release method initially dispenses a portion of the dose to swiftly achieve the effective therapeutic concentration of the drug (Li et al. 2018; Sreedharan and Singh 2019). As mentioned in the study by Sreedharan and Singh (2019), the initial burst release will serve as a loading dose to control disease dissemination, while the sustained‐release phase will enhance treatment efficacy (Sreedharan and Singh 2019). At the same time, the physicochemical properties of the particles are another parameter affecting the release profiles. For instance, in a study by Mercan et al. (2022), particle size was shown to affect the release kinetics in rifampicin‐loaded eudragit/polymer systems, and NPs with a smaller size provided a more controlled and more potent antibacterial effect. The present study found that the drug exhibited generally controlled release over a certain period, with a slight increase, followed by a stable profile. To elucidate the release mechanism, prevalent kinetic models (zero‐order, Higuchi, and Korsmeyer–Peppas) were employed. These models were chosen for their demonstrated efficacy in evaluating drug release from nanostructured and polymeric materials, thereby facilitating the distinction between diffusion‐controlled and combination mechanisms. The Higuchi model suggests that diffusion plays a significant role in controlling drug release from the formulation. On the basis of the *R* ^2^ values, the Korsmeyer–Peppas model (*R* ^2^ = 0.94386) provided the best fit to the experimental data, followed by the Higuchi model (*R* ^2^ = 0.91744). The system most likely exhibited a diffusion‐dominated release, with contributions from surface desorption of drug molecules initially associated with the particle surface, given the comparatively low release rate constant and the progressive release profile observed in the data. This may indicate that the release mechanism is predominantly diffusion‐controlled. The findings may indicate that nanoformulation facilitates a controlled and prolonged release of AMP, thereby improving therapeutic efficacy. While smaller particles or surface‐associated drugs may be responsible for the initial burst release seen in the release profile, larger or aggregated particles can slow down diffusion pathways, contributing to the persistent release phase (Bai et al. 2022). Such as PDI value (0.68) nevertheless indicates the presence of aggregation, which is commonly reported in NP‐based drug delivery systems, and this heterogeneity may also influence the drug release behavior.

The antimicrobial efficacy of both Amp and l‐glu–Fe_3_O_4_–Amp NPs was initially assessed against two widely utilized representative strains. Amp and l‐glu–Fe_3_O_4_–Amp NPs showed antibacterial property against both *B. cereus* and *S. aureus* in a concentration‐dependent manner. The ability of an antimicrobial agent to diffuse through a solid agar matrix is primarily assessed using the agar well diffusion method, the first screening method for materials. It is significantly impacted by physicochemical characteristics, such as hydrophilicity, size, and surface charge. In contrast, the MIC assay is conducted in broth, which minimizes diffusion restrictions and facilitates more direct interaction between the compounds and bacterial cells. Therefore, we concluded that the nanoformulation might have resulted in smaller inhibition zones than free antibiotics due to its particle size and decreased diffusion capacity. In addition, bacterial growth was monitored in the presence of Amp alone or l‐glu–Fe_3_O_4_–Amp NPs over time. As is well known, bacterial growth occurs in early, middle, and late log phases. Exposure to the environment occurs when bacteria adapt and begin reproducing; the late log phase is the point at which bacteria consume nutrients in the environment and enter a stationary phase. After this incubation period, bacterial growth stops (Rolfe et al. 2012). In this way, the aim was to observe how long the bacteria could continue to grow in the presence of the test materials. In this study, the growth kinetic experiments of *B. cereus* and *S. aureus* were performed in the absence and presence of different concentrations of Amp or l‐glu–Fe_3_O_4_–Amp NPs alone. The results indicated that, in comparison to the negative control, significant growth inhibition against both bacteria (especially against *S. aureus*) was observed for the selected concentrations of l‐glu–Fe_3_O_4_–Amp NPs or the commercial concentration of Amp. In a study (Rashid et al. 2021), Fe_3_O_4_ magnetic NPs were conjugated with vancomycin via dopamine, and the growth kinetics of *Bacillus subtilis* and *Escherichia coli* in the presence of the prepared formulation were analyzed, as in this study. In a different research (J. S. Kim et al. 2007), the growth kinetics of *Pseudomonas aeruginosa* were examined in the presence of silver NPs conjugated with gentamicin, and it was determined that the formulation caused a change in log phase after the sixth hour. In this study, both the drug Amp and its nanoantibiotic formulation exhibited growth inhibition against *B. cereus* after the first hour, whereas their inhibition effects against *S. aureus* became apparent after the second hour.

In the literature, it has been documented that the antimicrobial activity can be explained by different mechanisms, including the generation of ROS, release of metal ions, damage to the cell wall, and disruption of the cell membrane (Godoy‐Gallardo et al. 2021; Franco et al. 2022; Wang et al. 2017). However, the underlying mechanisms remain inadequately explored and require more comprehensive assessment. The interaction between NPs and bacterial membranes is mostly attributed to electrostatic interactions at the NP‐bacteria contact (Arakha et al. 2015; Behera et al. 2019). The antibacterial activities against Gram‐positive strains can be attributed to the release of ROS resulting from the binding of the nanoconjugate to the bacterial cell surface (Leena Panigrahi et al. 2023). This study aimed to examine whether the antimicrobial effectiveness of l‐glu–Fe_3_O_4_–Amp NPs was related to its ROS‐generating potential. The incubation time is selected to ensure that the effects of the test sample on the oxidative stress response are properly evaluated. This period covers the time when bacteria complete their logarithmic growth phase, and any adaptation to stress conditions or cellular damage mechanisms becomes visible. Additionally, a 24‐h incubation allows assessment of sustained oxidative stress rather than short‐term transient ROS fluctuations (Mammari et al. 2022). The assays elucidated that the application of l‐glu–Fe_3_O_4_–Amp NPs exhibited higher fluorescence intensity, namely, more ROS accumulation in both bacteria (especially in *S. aureus)* when compared with Amp.

The LIVE/DEAD BacLight fluorescent assay was used to differentiate viable from nonviable cells resulting from membrane damage. Certain integral membrane cells remain viable and exhibit green fluorescence upon treatment with the membrane‐permeable SYTO9 dye, while nonviable bacterial cells with compromised membranes display red fluorescence due to staining with PI. The small size of bacterial cells and their low scattering signal make it difficult to differentiate them from debris, cell fragments, and aggregates, directly affecting the gating process. This often leads to an underestimation of the percentage of viability, particularly in the control group. For instance, in an earlier study, the anti‐biofilm effectiveness of nanoformulation consisting of nano‐chitosan and CeO_2_ NPs against *P. aeruginosa* and *S. aureus* biofilms was evaluated according to the LIVE/DEAD BacLight fluorescent assay. The results exhibited that red fluorescence intensity increased in biofilms exposed to nanoformulation, clarifying that *the* nanoformulation causes the damages in biofilm structures (Uzair et al. 2020). In this study, using LIVE/DEAD BacLight assay enabled a much more quantitative data of bacterial cell viability after treatment of Amp alone or l‐glu–Fe_3_O_4_–Amp NPs. It was seen that both Amp and the l‐glu–Fe_3_O_4_–Amp NPs were more effective on *S. aureus* than *B. cereus* in terms of live and dead cells percentages. This result may be attributed to variations in cell wall composition and the bacteria's capacity to absorb charged particles (Khater et al. 2020).

The assessment of safety is crucial when contemplating the clinical use of any innovative nanodrug formulations. Biocompatibility tests are essential to demonstrate that nanoantibiotic delivery systems are not only effective but also safe for human health. These tests constitute the safety barrier in the transition from system design to clinical application. Cytotoxicity tests are the primary method for assessing whether nanocarriers, when used correctly, cause damage to cell health. Here, we assessed the potential toxicities of Amp alone and l‐glu–Fe_3_O_4_–Amp NPs alone. The obtained results uncovered that l‐glu–Fe_3_O_4_–Amp NPs, even at high concentrations, cause no significant toxicity after 24 h incubation, while Amp reduced cell viability in a dose‐dependent manner. The particle size, surface charge, material structure, and surface modification are critical factors that affect the toxic effects of NPs on cells (Ciappellano et al. 2016). This study showed that the nanoantibiotic formulation with an average particle size of 164 nm exhibited no significant cytotoxicity, indicating favorable biocompatibility within the permissible nanoscale range for drug delivery systems. The particle size facilitates effective cellular absorption while reducing possible toxicity, making the formulation a viable candidate for subsequent further in vivo assessments.

The intracellular localization of NPs is further confirmed by TEM image studies in the literature. TEM studies make it easier to observe morphological changes that follow a surface‐modification phase (Reifarth et al. 2018). For instance, the intracellular absorption of three Paclitaxel formulations in several human cancer cell lines was examined and compared by the authors using TEM (Madaan et al. 2013). Researchers investigated the impact of a PLGA NP nanoformulation on bacteria using TEM, with mycobacterial mycolic acid as a targeting moiety (Lemmer et al. 2015). In this study, TEM analysis showed that cell membrane integrity was disrupted, and electron‐dense regions were significantly redistributed in cells treated with l‐glu–Fe_3_O_4_–Amp NPs. These findings indicate that the nanoantibiotic formulation increases permeability by targeting the cell membrane, it was effectively taken up by the cell, leading to an abnormal contrast distribution in the cytoplasm. The interaction of the nano‐dye‐labeled formulation with bacterial cells was also investigated using confocal microscopy. These findings, when considered together with the membrane integrity disruption and cytoplasmic changes observed in TEM analyses, reveal that the dye labeled‐l‐glu–Fe_3_O_4_–Amp NPs effectively penetrate bacterial cells and can localize within the cellular environment.

Molecular docking results suggest that the antibacterial activity of the l‐glu–Fe_3_O_4_–Amp NPs formulation is mediated through direct interaction with PBP1a, a key enzyme localized at the bacterial cell wall and essential for peptidoglycan biosynthesis in Gram‐positive bacteria, such as *S. aureus* and *B. cereus* (Sauvage et al. 2008). The observed high binding affinity (−9.3 kcal/mol) and the presence of a sufficiently large and accessible binding cavity (1711 Å^3^) within this simplified in silico model indicate that the Amp moiety remains potentially available for target engagement despite conjugation to magnetic NPs. To further support this interpretation, free Amp was docked to PBP1a under identical conditions and exhibited a comparable binding affinity (−8.8 kcal/mol) with highly overlapping contact residues. This comparative analysis suggests that the formulation does not reduce Amp's intrinsic binding capability to PBP1a. The involvement of polar residues, such as Ser, Thr, Lys, and Arg, together with hydrophobic interactions mediated by aromatic residues, reflects a binding pattern consistent with established β‐lactam–PBP recognition. These findings may indicate that the formulation retains the potential to interact with PBPs, thereby contributing to bacterial cell wall synthesis and explaining the observed antibacterial behavior. Moreover, the slightly more favorable binding affinity of l‐glu–Fe_3_O_4_–Amp NPs compared with free Amp suggests that NP formulation may contribute to modest stabilization of the ligand–protein complex without altering the canonical β‐lactam binding mode. This mechanistic interpretation is fully consistent with a recently published study (Elabed et al. 2025), which demonstrated that amoxicillin‐conjugated magnetic NPs retained stable binding to PBP1a and that NP conjugation did not compromise target specificity. In line with the results of earlier studies, the present results suggested that the antibacterial efficacy of l‐glu–Fe_3_O_4_–Amp NPs is primarily associated with preserved PBP‐mediated interactions rather than nonspecific NP–bacteria associations, thereby providing supportive insight into bacteria–formulation interaction through a well‐established β‐lactam mechanism, while also being supported by experimental findings.

The findings of this study can be rationalized by bacterial cell viability decreasing with a decrease in the presence of l‐glu–Fe_3_O_4_–Amp NPs in both strains, indicating clear interactions between NPs and bacteria. Examining the significance of potentials in interfacial interactions, it was observed that the efficacy was higher in *S. aureus* than in *B. cereus* strain.

## 5 Conclusion

Our current investigation has shown that Amp formulations are effective against bacteria compared with pure antibiotics. This finding further demonstrates the function of l‐glu–Fe_3_O_4_–Amp as a biocompatible drug delivery system, devoid of adverse effects on microbial functional activities and cell viability. In conclusion, the interaction of NPs at the bacterial interface is crucial to defining the antibacterial efficacy of metal oxide NPs, and this study highlighted the significance and future perspectives of metal oxide NPs in bacterial diagnosis and treatment.

## Author Contributions

**Mehmet Demirel:** conceptualization, methodology, investigation, data curation, writing – original draft. **Ozlem Baris:** methodology, investigation, validation. **Mesut Taskin:** methodology, investigation. **Seyda Albayrak:** methodology, formal analysis. **Ferhunde Aysin:** methodology, investigation. **Azada Aliyeva:** methodology. **Buket Bakan:** methodology, data curation, supervision, writing – original draft, writing – review and editing.

## Funding

The authors have nothing to report.

## Ethics Statement

The authors have nothing to report.

## Conflicts of Interest

None declared.

## Data Availability

The data used to support the findings of this study are included within the manuscript.
